# A critical review of absorption-driven CO_2_ capture and techno-economic feasibility

**DOI:** 10.1039/d6ra04204j

**Published:** 2026-09-11

**Authors:** Ghzzai Almutairi, Fekri Abdulraqeb Ahmed Ali, Salma A. Al-Zahrani, Norah Alwadai, S. Ganesan, Mohamed Alsaleh, Mohammed Asiri, Sammer Alnassar, T. Vinod Kumar, S. Padmanabhan, P. Saravanan, S. Jayakumar, C. Kavitha

**Affiliations:** a Hydrogen Technologies Institute, King Abdulaziz City for Science & Technology (KACST) Riyadh Saudi Arabia gmotari@kacst.gov.sa; b Chemical Engineering Department, College of Engineering, Imam Mohammad Ibn Saud Islamic University (IMSIU) Riyadh 11432 Saudi Arabia; c Chemistry Department, Faculty of Science, University of Ha'il P.O. Box 2440 Ha'il 81451 Saudi Arabia; d Department of Physics College of Science, Princess Nourah bint Abdulrahman University P.O. Box 84428 Riyadh 11671 Saudi Arabia; e Department of Mechanical Engineering, Sathyabama Institute of Science and Technology Chennai India; f Department of Mechanical Engineering, Vels Institute of Science, Technology and Advanced Studies (VISTAS) Chennai India; g Department of Mechanical Engineering, Vel Tech Rangarajan Dr. Sagunthala R&D Institute of Science and Technology Chennai 600062 India; h Department of Chemistry, St. Joseph's College of Engineering OMR-600119 Chennai India; i Department of Chemistry, Jerusalem College of Engineering (Autonomous) Pallikaranai 600100 Chennai India; j Department of Chemistry, Dwaraka Doss Goverdhan Doss Vaishnav College (Autonomous) (Affiliated to the University of Madras, Chennai) 833, Gokul Bagh, E.V.R. Periyar Road, Arumbakkam Chennai 600 106 Tamil Nadu India kavithakandhan@gmail.com

## Abstract

Rapid industrial expansion combined with continuous population growth has intensified global environmental concerns, particularly the increasing concentration of greenhouse gas (GHG) emissions that accelerate climate change. Addressing these challenges requires the implementation of sustainable and large-scale mitigation strategies, among which carbon capture technologies have emerged as a vital approach for reducing atmospheric CO_2_ emissions. Among the available capture techniques, absorption-based systems remain the most mature and widely adopted due to their high processing capacity, operational reliability, and adaptability across diverse industrial applications. This review critically examines recent advancements in CO_2_ capture technologies, with a primary emphasis on absorption-based processes. The review critically evaluates the techno-economic feasibility of absorption-based CO_2_ capture by comparing solvent selection strategies, regeneration energy requirements, and key economic indicators relevant to industrial implementation. In addition, the review compares absorption processes with other separation techniques, highlighting their advantages, limitations, and industrial relevance. Special attention is given to recent innovations in solvent development, including conventional aqueous amine solvents, phase-change solvents, and emerging deep eutectic solvents, which have demonstrated improved capture performance and enhanced process sustainability. Finally, the review identifies the major technical and economic challenges associated with absorption-based CO_2_ capture and proposes strategic directions to improve efficiency, reduce operational costs, and support large-scale industrial deployment.

## Abstract 2

Emerging CO_2_ capture strategies based on advanced solvents and separation technologies, supported by process optimization and techno-economic analysis, offer sustainable pathways for industrial emission reduction. Image created with the assistance of Microsoft Copilot.

## Graphical abstract

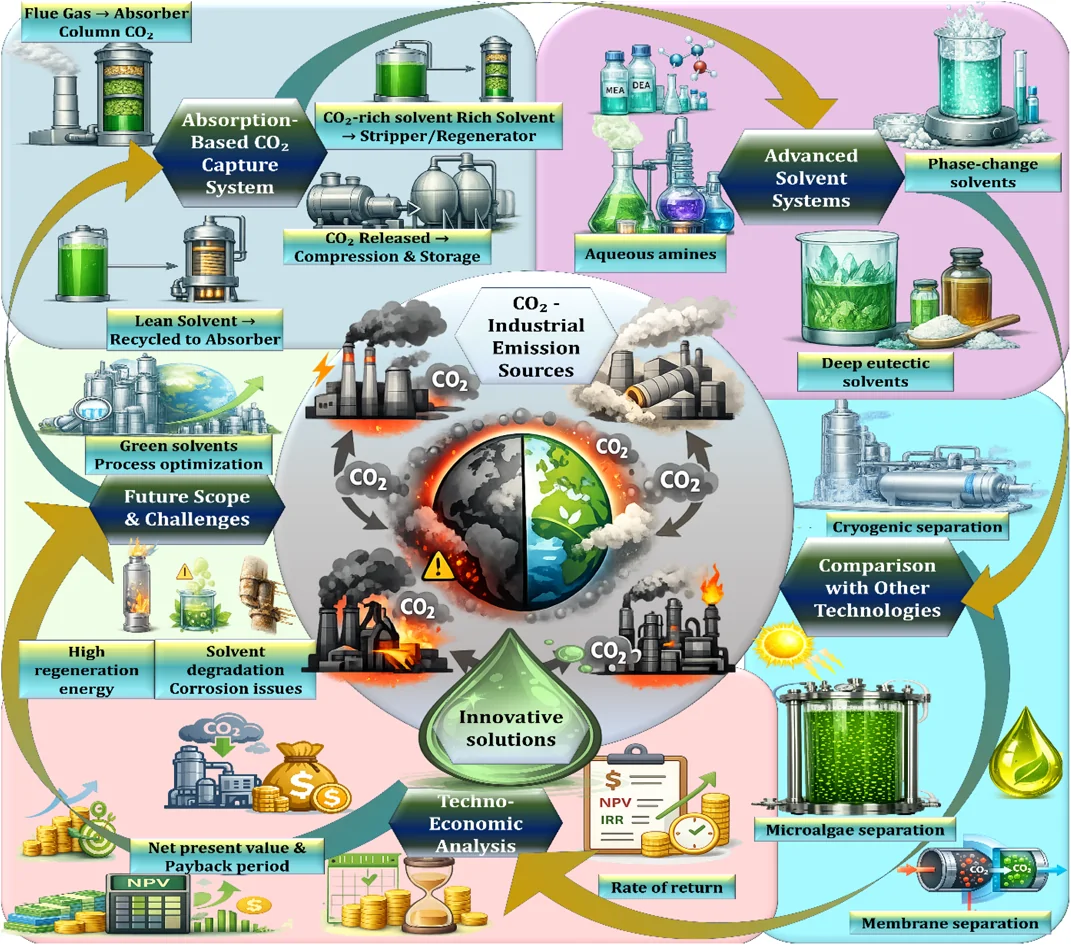

## 1 Introduction

Greenhouse gases (GHGs), most notably carbon dioxide (CO_2_) have been increasing to unprecedented levels in the atmosphere over the past few decades, escalating environmental degradation and causing climate change on a planetary scale.^1^ This has led to a broad range of climatic disruptions like the rise in the sea level, abnormal rainfall patterns, severe weather conditions such as storms, and extended droughts. According to recent climate assessments, the global average surface temperature has increased by approximately 1.3–1.5 °C above the pre-industrial baseline, largely due to anthropogenic greenhouse gas emissions, with atmospheric CO_2_ concentrations continuing to increase annually.^2^ One of the major drivers of global warming is the continuous rise in atmospheric CO_2_ concentration, which has increased by more than 50% since the pre-industrial era, from approximately 280 ppm to over 430 ppm in 2025–2026, representing the highest level recorded in modern human history.^3^ This growth has mainly been attributed to the extensive burning of fossil fuels and land-use activities, including deforestation and urban sprawl. Besides causing changes in the global temperatures, high levels of CO_2_ have caused changes in ocean chemistry as well due to the rise in acidity by almost 30% (ref. 4). This pH change has severe implications on the marine ecosystem including negative impacts on the biodiversity and endangering the survival of numerous aquatic organisms.^5^

Recent global energy assessments indicate that energy demand continues to increase despite the rapid deployment of renewable energy technologies. According to the International Energy Agency (IEA), global energy demand increased by approximately 2.2% in 2024, driven primarily by growth in electricity consumption, industrial activity, cooling demand, and electrification. Although renewable energy sources expanded significantly, fossil fuels continue to account for the majority of global energy consumption. Consequently, energy-related CO_2_ emissions reached a record level of approximately 37.8 Gt in 2024, highlighting the continuing dependence on fossil fuels and reinforcing the urgent need for efficient carbon capture, utilization, and storage (CCUS) technologies to achieve global climate targets.^6–8^

The increasing severity of CO_2_ emissions has increased the necessity of an overall shift to cleaner energy systems and sustainable industrial procedures. Carbon capture, utilization, and storage (CCUS) is one of the viable and practical solutions to CO_2_ emissions reduction in this transitional stage.^5^ Based on the projections of the International Energy Agency (IEA), it is estimated that carbon capture and storage (CCS) could contribute about 20% of the necessary greenhouse gas (GHG) emission mitigation by the year 2050.^9^

Carbon capture technologies are broadly classified into three categories: pre-combustion, oxy-fuel combustion, and post-combustion (Fig. 1).^10^ As an example of post-combustion CO_2_ capture, Mukherjee *et al.* performed a techno-economic analysis of activated carbon-based CO_2_ capture and assessed various precursor materials for activated carbon production.^10^ In pre-combustion systems, the fuel is separated of CO_2_ before the combustion phase. In comparison, post-combustion capture includes the removal of CO_2_ in the flue gases following the combustion of fuel, and it uses a variety of methods to capture it: cryogenic separation, membrane-based separation, adsorption process, chemical looping and physical and chemical absorption.^11^ Oxy-fuel combustion, in its turn, involves burning fuel with pure oxygen, rather than air, but recirculated flue gas is employed to regulate the burn temperature. The result of this process is an exhaust stream that consists mainly of CO_2_, and water vapor, enabling the recovery of CO 2 to be effectively achieved by condensing water.^12^

**Fig. 1:**
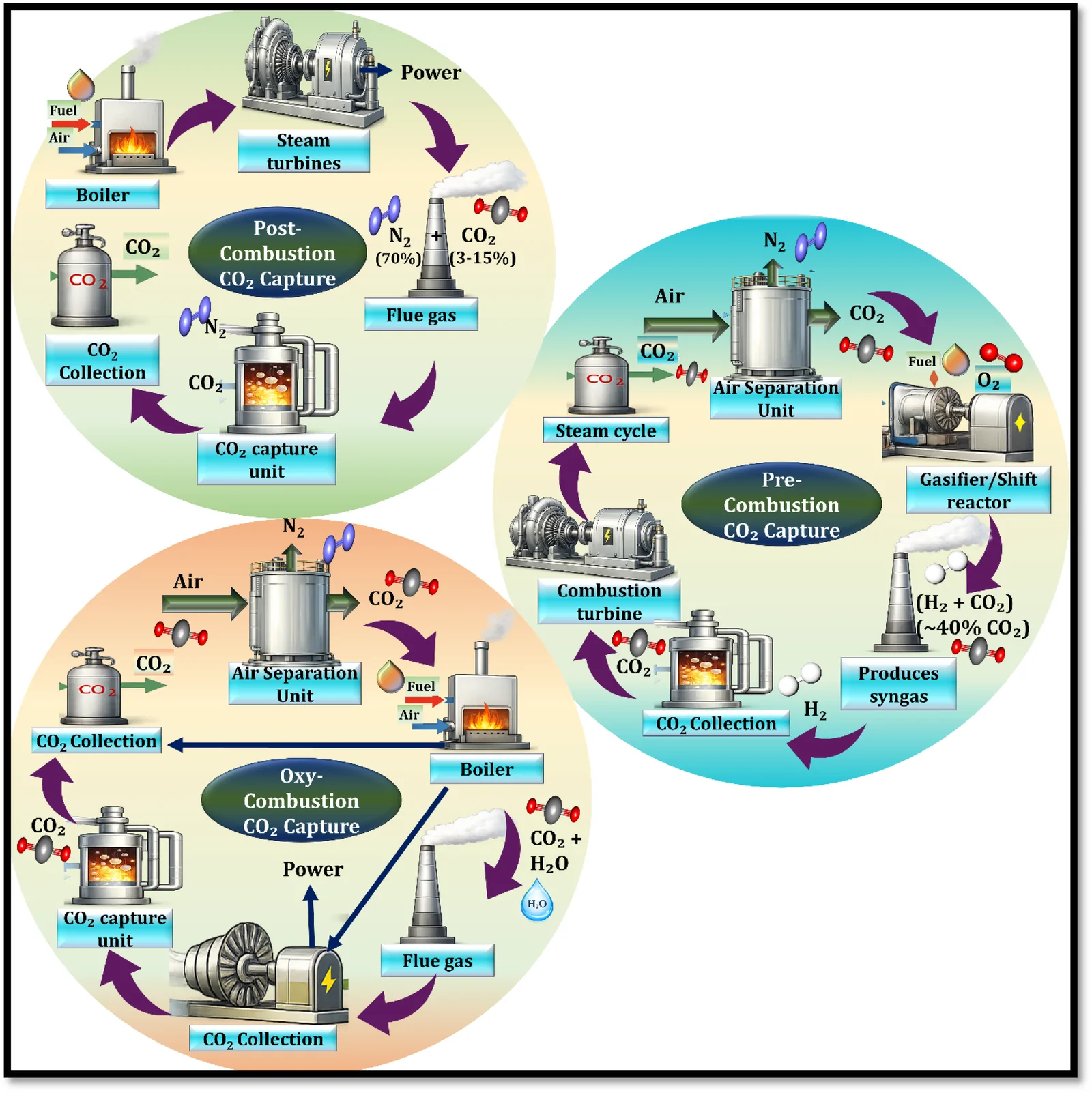
Schematic representation of carbon dioxide capture methods. Reproduced with permission from ref. 10 Copyright © Elsevier. License Number: 6298750753961.

The practical challenges and operational benefits in the capture pathways of carbon differences greatly differ. High CO_2_ partial pressures and high-enriched gas streams in pre-combustion capture, enhance separation to enhance the performance of the absorption process, reducing the solvent requirement.^10^ Nevertheless, this approach relies on upstream gasification of the fuel, which adds new processing steps, adds complexity to the system, and increases costs. Post-combustion capture, however, is much more appropriate to use in the existing industrial and power generation plants since it can be applied without significant changes to the existing infrastructure. This renders it a viable alternative in emission reduction in the short-term. However, the low concentration of CO_2_ in flue gases and the presence of impurities in it impairs its performance and can lower the capture efficiency and result in solvent degradation.^13^ Oxy-fuel combustion is a different approach where fuel is burnt in an oxygen-abundant environment, to form a flue gas stream that is mainly made up of CO_2_. This greatly facilitates the separation process and the capture efficiency is increased greatly. This advantage necessitates the large scale generation of oxygen that incurs a huge energy penalty on the process and makes the process less economic.

Absorption has been considered to be one of the most dependable and well-used methods of capturing CO_2_ mainly because it enjoys greater flexibility of operation and is compatible with various types of industrial set up such as pre-combustion, post-combustion and oxy-fuel capturing. As much as adsorption and absorption technologies have been extensively researched, specific studies that have focused on absorption processes are relatively scarce. In a different study, Singh and Dhar looked into microalgae-based systems as a viable way of CO_2_ sequestration.^14^ It has also had environmental impact assessment to determine absorption-based CO_2_ capture in post-combustion flue gas treatment that offers insights on its ecological implications.^15^ Also, Podder *et al.* offered a general overview of the carbon capture and utilization methods, such as absorption approaches.^16^ The recent developments in absorption technology have mainly concentrated on the energy efficiency issues especially in the regeneration of solvents.^17^ Addition of catalysts has been reported to greatly decrease the regeneration energy needs, which in turn enhances efficiency of the entire process. Furthermore, sepiolite clay was also found to be a good support material in Fe_2_O_3_ and CUO catalysts, and enhance the CO_2_ desorption performance.^18^ Studies He *et al.* in solvent blends have shown better capture efficiency and less energy penalty with dual-amine blends.^19^ Moreover, Zhang *et al.* have also referenced an emergent solvent system like phase-change solvents and non-aqueous media, which have been critically assessed and their advantages and existing limitations to CO_2_ capture use identified.^20^ Although absorption-based CO_2_ capture is well known due to its technological maturity and flexibility in a variety of industrial applications, a thorough analysis of its recent progress, in particular, a detailed techno-economic perspective, is not very widespread. It is important to conduct a detailed evaluation of the economic viability and environmental performance to reinforce its practical implementation and sustainable performance in the long run.

The present research provides a comprehensive overview of carbon capture technologies, with particular emphasis on absorption processes. It traces how absorption-based CO_2_ capture has evolved, highlights recent progress, and discusses the persistent challenges linked to solvent selection, process efficiency, and operational constraints. In contrast to previous reviews that either focus mainly on solvent chemistry or on high-level CCUS techno-economic assessments, this work brings together solvent properties, process design, and detailed techno-economic analysis within a single, integrated framework that is specifically tailored to absorption-driven CO_2_ capture. A central contribution of this review is the introduction of a unified solvent comparison framework that links CO_2_ absorption performance (capacity, kinetics, cyclic stability, degradation and loss) directly to regeneration energy penalties and key economic indicators, enabling process-relevant ranking of solvents rather than purely material-centric comparison. In doing so, it sets out clear procedures for conducting techno-economic studies, focusing on the variables that most strongly influence cost, energy use, and capture performance. The findings underline the need for more robust and standardized evaluation frameworks, supported by close collaboration between engineers, economists, and environmental scientists. Developing such integrated approaches will be essential to improve cost-effectiveness, refine system design, and enable the large-scale deployment of absorption-based CO_2_ capture technologies.

## 2 Comprehensive survey of carbon dioxide capture technologies

Fig. 1 gives a general idea of the key technologies used to capture CO_2_, but the selected separation mechanisms of the technologies are emphasized in Fig. 2.^21^ The major techniques of separating CO_2_ include absorption, adsorption, membrane, algal, and cryogenic. Both of these methods share characteristic operational advantages and some drawbacks, and they are all tabularly summarized in Table 1.^17,22,23^ It highlights that no single CO_2_ capture technology is universally optimal. Adsorption, membranes, cryogenic, and algal systems each offer specific advantages but face material, energy, or scalability constraints, whereas absorption combines high CO_2_ purity and technological maturity with significant penalties in regeneration energy and solvent degradation. This contrast supports making absorption the main focus of the review, since it is already deployed at scale but still requires substantial techno-economic optimization.

**Fig. 2:**
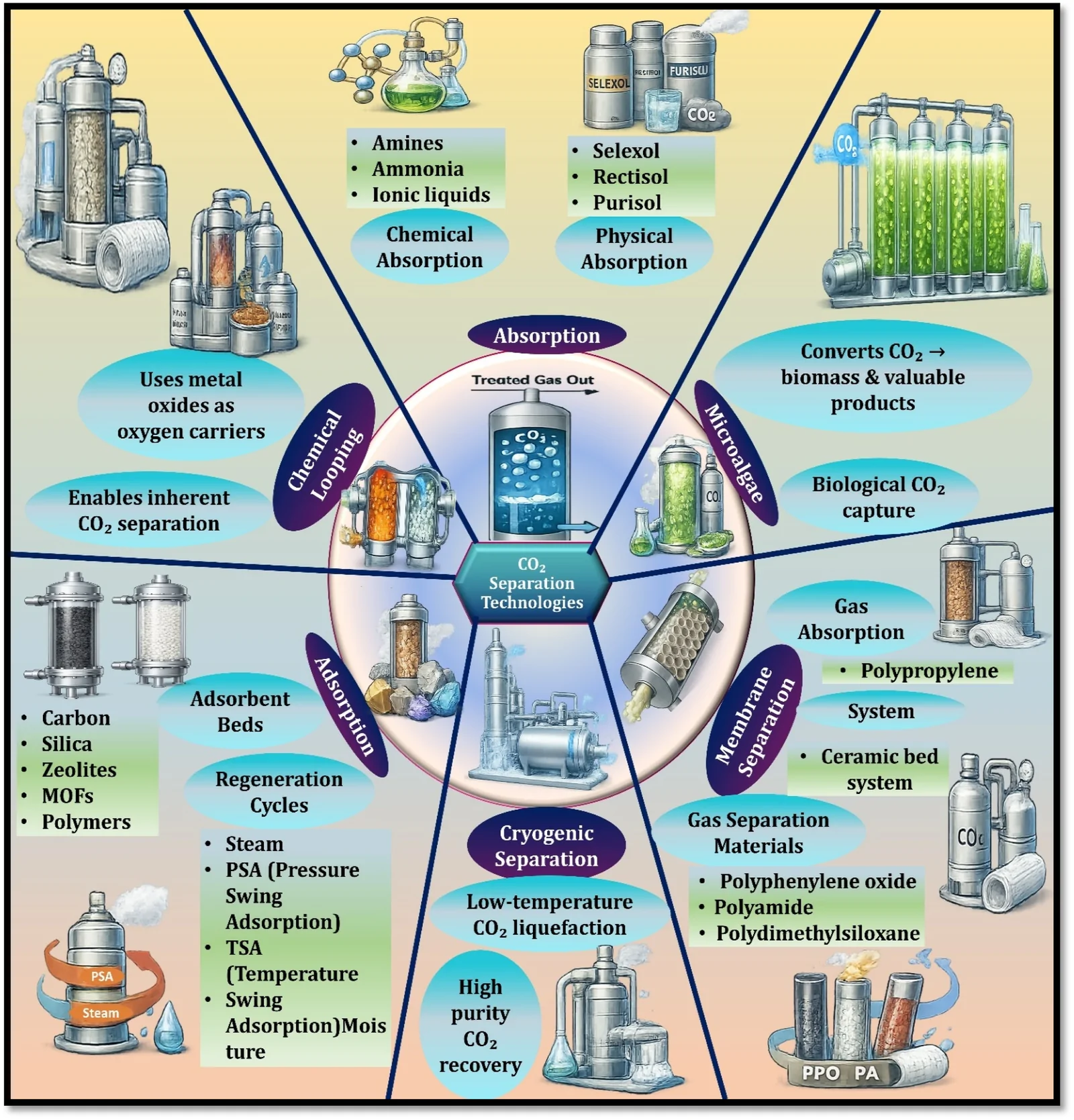
Comprehensive classification of CO_2_ separation technologies and their mechanistic pathways. Adapted from,^21^ with permission from Elsevier. Copyright © Elsevier. License number: 6298760708621.

**Table 1:** Comparative assessment of benefits and challenges in CO_2_ capture and separation methods.^17,22,23^

S.No	Technology	Key advantages	Key limitations
1	Adsorption	High CO_2_ purity; low regeneration heat; flexible (PSA/TSA); high efficiency	Costly adsorbents; regeneration needed; material development challenges
2	Absorption	Mature & proven; very high CO_2_ purity	Energy-intensive solvent regeneration; solvent degradation
3	Cryogenic	Very high purity; established technology	Very high energy demand; expensive; niche use
4	Algae systems	Converts CO_2_ to biomass; useful for wastewater; potentially low-cost	Large land needed; harvesting difficulty; variable efficiency
5	Membrane	Low energy; compact & modular; continuous operation	Fouling issues; requires high-performance membranes

### 2.1 Membrane-based carbon dioxide separation technologies

Membrane-based separation has attracted considerable attention as an alternative CO_2_ capture technology because of its relatively low energy consumption, compact design, modular configuration, and ease of integration with existing industrial processes. However, these advantages are often context-dependent and may diminish under conditions requiring high CO_2_ purity or recovery. Unlike absorption processes, membrane systems separate gases through selective permeation without requiring phase change or chemical regeneration, making them inherently simple and suitable for continuous operation. This simplicity, however, is offset by limitations in achieving both high selectivity and high permeability simultaneously. The separation efficiency primarily depends on differences in gas diffusivity and solubility within the membrane material, enabling preferential transport of CO_2_ over other gas components such as N_2_, CH_4_, and H_2_. Owing to these advantages, membrane technology has been investigated for applications including natural gas sweetening, hydrogen purification, biogas upgrading, and post-combustion CO_2_ capture.^23–26^

Significant advances in membrane materials have substantially improved separation performance during the past decade. Conventional polymeric membranes remain the most commercially developed because of their relatively low fabrication cost, ease of processing, and good mechanical flexibility. However, their performance is often restricted by the well-known permeability–selectivity trade-off and susceptibility to plasticization under high CO_2_ pressures. This trade-off represents a fundamental material limitation, constraining further performance improvements without compromising one parameter over the other. To overcome these limitations, considerable research has focused on mixed-matrix membranes (MMMs), which incorporate inorganic fillers such as zeolites, silica nanoparticles, or metal–organic frameworks (MOFs) into polymer matrices to simultaneously enhance permeability and selectivity. Nevertheless, issues such as poor interfacial compatibility, filler agglomeration, and defect formation often limit the practical performance of MMMs compared to their theoretical potential. Likewise, ceramic membranes offer excellent thermal and chemical stability, making them suitable for high-temperature industrial applications, whereas MOF-based membranes provide highly tunable pore structures and exceptional CO_2_ adsorption characteristics, enabling improved gas separation efficiency under moderate operating conditions.^27–31^ Despite these promising attributes, the scalability and mechanical robustness of MOF-based membranes remain significant challenges for industrial deployment. The major advantages, limitations, and typical industrial applications of these representative membrane technologies are summarized in Table 2. As shown in Table 2, commercially mature polymeric and thin-film composite membranes provide moderate CO_2_ permeabilities and selectivities at relatively low cost, while more advanced systems such as mixed-matrix, zeolite, carbon molecular sieve, MOF-based, and graphene membranes can reach much higher selectivities or permeabilities but are constrained by issues including interfacial defects, brittleness, moisture sensitivity, and complex large-scale fabrication. Facilitated transport and ionic liquid membranes exhibit extremely high CO_2_/N_2_ selectivities under mild operating conditions, yet carrier degradation and stability concerns still limit long-term operation. Overall, the data in Table 2 indicate that membrane processes tend to be most attractive when compactness, modularity, and high flux are prioritized, whereas absorption offers more robust performance and better-understood economics for large-scale post-combustion CO_2_ capture.

**Table 2:** Performance evaluation of membrane processes for CO_2_ separation

S.No	Membrane type	Synthesis method	Temperature (°C)	CO_2_ permeance (GPU)	Pressure (kPa)	CO_2_/N_2_ selectivity	Key findings	Ref.
1	Bipolar membrane	*In situ* mineralization	—	—	—	—	• The membrane captured 60–85% of dissolved inorganic carbon, with only minor Mg(OH)_2_ precipitation	24
							• Optimizing current density and residence time can substantially lower energy consumption	
2	Highly selective hollow fiber membranes (HF)	*In situ* layer-by-layer (LbL) surface functionalization	—	8341 GPU	—	1.1	• Polymer membrane achieved CO_2_ : N_2_ selectivity of 240 with 1018 Barrer CO_2_ permeability (101 GPU permeance)	25
							• Surface functionalization enhanced hydrophilicity and enabled layer-by-layer deposition	
3	PDMS & MOF membrane	Simple coordination modulation method	308 K	10 450	100	9.1	• Amorphous MOF nanosheets improve TFC polymeric membranes, enhancing CO_2_/N_2_ separation, permeability, and processability	26
4	Double-layered Pebax-1657/PDMS nanomembranes	Spin coating	298 K	1200–3500	300	23–72	• CO_2_-selective Pebax-1657 on a PDMS gutter layer enhanced CO_2_/N_2_ selectivity through nanoscale blending, outperforming pristine Pebax-1657 for post-combustion CO_2_ capture	27
5	Continuous zeolitic imidazolate framework (ZIF-8) membranes on titania-functionalized porous polymeric supports	—	293 K	—	100	H_2_/CO_2_ = 7 : 1	• Ultrathin ZIF-8 membranes on titania-functionalized polymer supports showed high H_2_ permeance and H_2_/CO_2_ selectivity for gas separation and sensing	28
6	Zeolitic imidazolate framework-8 (ZIF-8)/graphene oxide (GO) membrane	Secondary growth method	298 K	104	100	7	• Defect-free ZIF-8/GO membrane with hybrid nanosheet seeding enabled rapid crystal intergrowth and high CO_2_/N_2_ selectivity, offering potential for other MOF and zeolite membranes	29
7	Polycrystalline metal–organic framework (MOF) membranes	—	303 K	120–330	100	27.2–37.3	• Rapid heat treatment (RHT) enhances CO_2_ capture and gas separation performance	30
							• Achieves high CO_2_/CH_4_, CO_2_/N_2_, and H_2_/CH_4_ selectivities with complete C_3_H_6_ rejection	
							• Improved separation arises from increased lattice stiffness without altering crystallinity	
8	High-performance ultrathin film composite (UTFC) membrane	Physical coating	25 °C	2860	0.2 MPa	28.2	• Copper hydroxide nanofibers enable ultrathin selective layers for CO_2_ capture	31
							• Achieves 2860 GPU CO_2_ permeance, 28.2 CO_2_/N_2_ selectivity, and 2.5-fold higher permeance	
							• Outperforms state-of-the-art polymer membranes	

Despite these technological developments, several challenges continue to hinder the widespread commercial deployment of membrane-based CO_2_ capture systems. One of the primary limitations is membrane fouling and degradation caused by contaminants commonly present in industrial flue gases, including sulfur oxides (SO_*x*_), nitrogen oxides (NO_*x*_), particulate matter, and water vapor. These impurities can reduce membrane permeability, deteriorate separation performance, and shorten membrane lifetime. This necessitates extensive gas pretreatment, which adds complexity and partially offsets the inherent simplicity of membrane systems. In addition, membrane systems frequently require high feed pressures or multiple membrane stages to achieve high CO_2_ recovery and purity, thereby increasing energy consumption and capital costs. As a result, the perceived low-energy advantage of membrane processes may not be fully realized in large-scale applications. The need for gas compression further contributes to operational expenses, particularly for post-combustion applications where the CO_2_ partial pressure is relatively low. Moreover, the long-term stability, large-scale fabrication, and economic viability of many advanced membrane materials, including MOF-based and facilitated transport membranes, remain under active investigation.^32–34^ This indicates a critical gap between material innovation and process-level implementation, where many promising membranes fail to meet industrial reliability and cost requirements over extended operation.

From a critical perspective, membrane technology offers several operational advantages, particularly its modularity, compact footprint, and relatively low maintenance requirements. Nevertheless, its industrial implementation remains limited when compared with absorption-based CO_2_ capture. Most reported high-performance membrane systems have been evaluated under laboratory or pilot-scale conditions using controlled gas compositions, whereas industrial flue gases exhibit significant variations in temperature, pressure, moisture content, and contaminant concentration that may adversely affect membrane performance. Furthermore, differences in membrane materials, testing protocols, operating conditions, and performance metrics among published studies make direct comparison difficult and often hinder objective evaluation of their practical applicability. Consequently, although membrane separation represents a promising complementary technology, particularly in hybrid CO_2_ capture systems, absorption-based processes continue to dominate commercial carbon capture owing to their higher technological maturity, superior capture efficiency, operational flexibility, and successful large-scale deployment in power plants and industrial facilities (Fig. 3). Therefore, the subsequent sections of this review primarily focus on recent advances in absorption-based CO_2_ capture, solvent development, process intensification, desorption enhancement strategies, and techno-economic feasibility, which constitute the principal scope of this review.

**Fig. 3:**
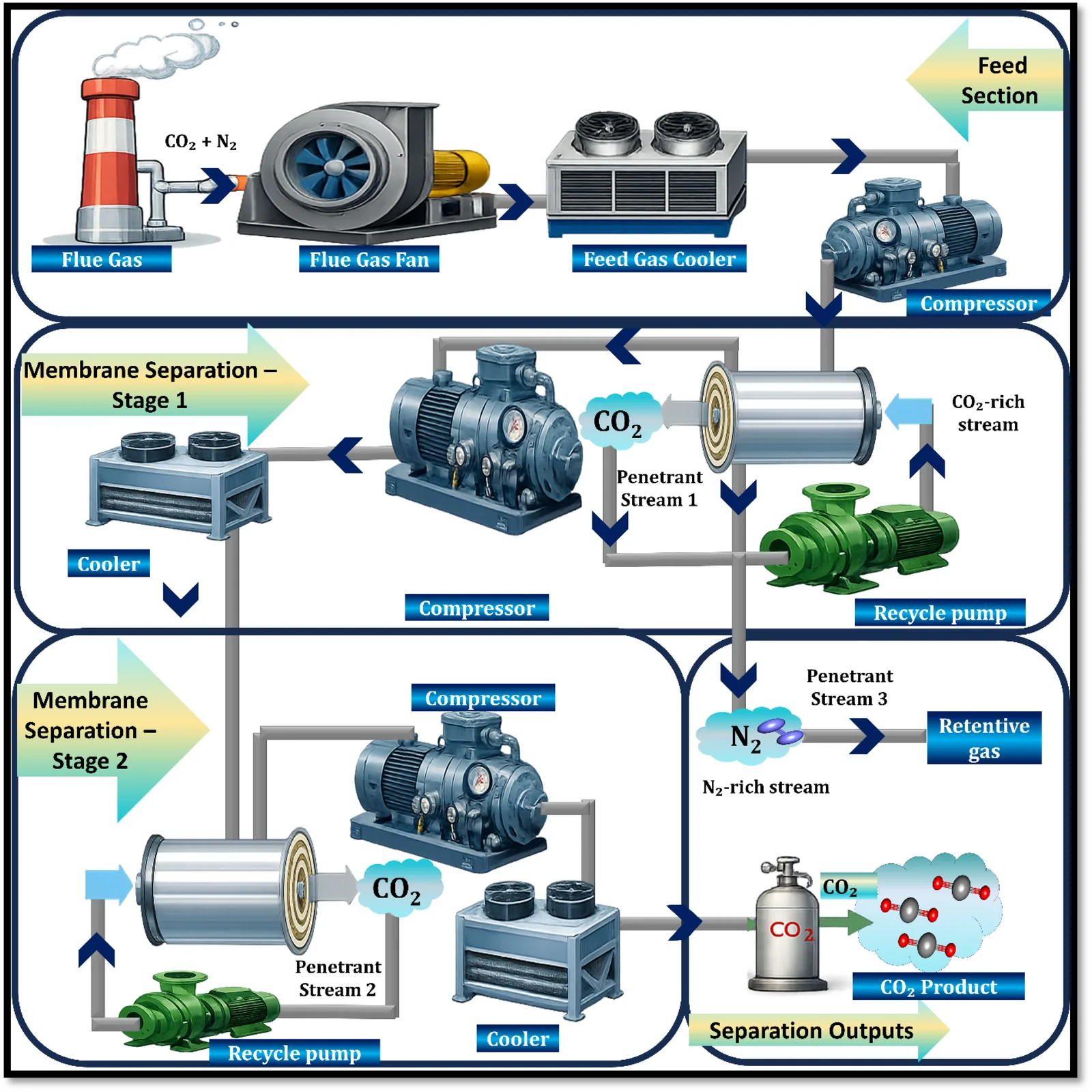
Schematic illustration of a membrane separation system for CO_2_ recovery from flue gas incorporating permeate recycling. Reproduced from ref. 34, Copyright © 2020 Elsevier, distributed under the Creative Commons CC BY 4.0 License.

### 2.2 Cryogenic techniques for carbon dioxide capture and purification

Cryogenic separation is a physical CO_2_ capture technology that exploits differences in the phase-transition temperatures of gas components to achieve high-purity carbon dioxide recovery. The process involves cooling gas mixtures to very low temperatures, causing CO_2_ to condense or desublimate into a liquid or solid phase while the remaining gases remain in the gaseous state. Unlike absorption processes, cryogenic separation does not require chemical solvents and therefore avoids issues associated with solvent degradation, corrosion, and chemical waste generation. Consequently, cryogenic technology has been considered particularly attractive for gas streams containing relatively high CO_2_ concentrations, where high-purity CO_2_ recovery is required for subsequent utilization or geological storage.^35,36^

The effectiveness of cryogenic separation depends primarily on the thermodynamic behaviour of individual gas components, including their boiling points, condensation temperatures, and sublimation characteristics. Before entering the cryogenic unit, flue gases generally require pretreatment to remove contaminants such as sulfur oxides (SO_*x*_), nitrogen oxides (NO_*x*_), moisture, and particulate matter that could freeze under cryogenic conditions and adversely affect process performance or damage equipment. Following purification, the gas stream undergoes successive cooling stages until CO_2_ is selectively condensed or deposited as a solid, while lighter gases such as nitrogen remain in the vapour phase. The recovered CO_2_ is subsequently reheated or pressurized to obtain a concentrated product suitable for transportation, storage, or utilization.^35–37^

Several cryogenic process configurations have been developed to improve separation efficiency and reduce energy consumption. Conventional cryogenic distillation columns remain widely employed for gas purification, whereas packed-bed cryogenic systems have attracted increasing attention because they facilitate direct CO_2_ deposition onto cooled packing materials or heat-exchanger surfaces. In these systems, the deposited CO_2_ is periodically recovered through controlled heating or pressure variation, enabling repeated capture–regeneration cycles. Fig. 4 illustrates a typical packed-bed cryogenic separation system, highlighting the principal stages involved in gas cooling, CO_2_ solidification, recovery, and regeneration. Such configurations have demonstrated high CO_2_ recovery efficiencies and product purities, particularly under high-pressure operating conditions.^36–39^

**Fig. 4:**
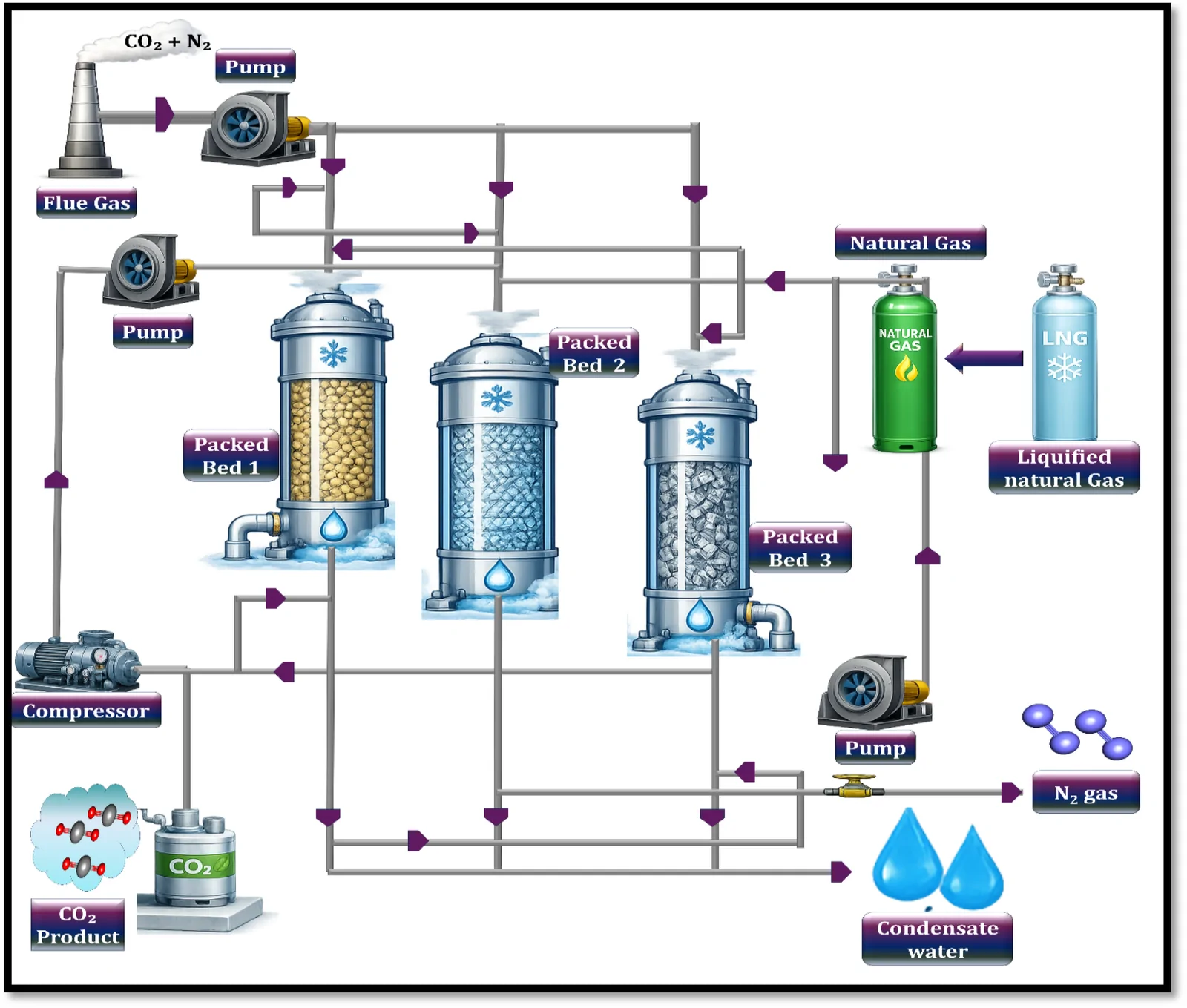
Detailed illustration of a packed-bed configuration for cryogenic separation of CO_2_ at reduced temperatures. Adapted from ref. 36, with permission from Elsevier. Copyright © Elsevier. License number: 6298770244977.

Despite these advantages, cryogenic CO_2_ capture remains one of the most energy-intensive carbon capture technologies because substantial refrigeration duty is required to achieve and maintain extremely low operating temperatures. The associated cooling infrastructure considerably increases both capital investment and operating costs, making the technology less attractive for dilute post-combustion flue gases. Furthermore, operational challenges including frost formation, equipment complexity, heat integration requirements, and process control under fluctuating industrial conditions continue to limit large-scale implementation. Although recent developments in cryogenic process design and heat recovery have reduced some of these energy penalties, the overall economics remain less favourable than those of mature absorption-based technologies.^35,39^

From a critical perspective, cryogenic separation offers distinct advantages for applications requiring very high CO_2_ purity, particularly in natural gas processing, liquefied natural gas production, and high-pressure industrial gas streams. However, most reported studies have been conducted under controlled laboratory or pilot-scale conditions, and their economic feasibility strongly depends on feed gas composition, operating pressure, refrigeration efficiency, and process integration. In comparison, absorption-based CO_2_ capture provides greater operational flexibility, lower overall energy consumption for dilute flue gases, and significantly higher technological maturity for large-scale industrial deployment. Therefore, although cryogenic separation remains an important complementary technology for specialized applications and hybrid carbon capture systems, absorption-based processes continue to represent the most practical and commercially established approach for post-combustion CO_2_ capture, which is the primary focus of this review.

### 2.3 Microalgal pathways for sustainable CO_2_ capture and utilization

Microalgae-based carbon capture has emerged as an environmentally sustainable approach for mitigating atmospheric CO_2_ while simultaneously producing valuable biomass. Unlike conventional physicochemical separation technologies, microalgae utilize photosynthesis to assimilate CO_2_ into cellular biomass using solar energy, thereby converting greenhouse gases into renewable resources. During cultivation, CO_2_-rich gas streams are introduced into algal cultures, where dissolved inorganic carbon is assimilated through photosynthetic pathways to produce carbohydrates, lipids, proteins, and other bioactive compounds, enabling combined carbon sequestration and biomass production for applications such as biofuels, wastewater treatment, animal feed, and high-value biochemicals.^1,37^

The effectiveness of microalgal CO_2_ capture depends on several operational parameters, including species selection, light intensity, nutrient availability, CO_2_ concentration, temperature, and reactor configuration. Many microalgal species possess efficient carbon-concentrating mechanisms that enable effective CO_2_ fixation even at relatively low atmospheric concentrations, while higher CO_2_ concentrations available from industrial flue gases can significantly enhance biomass productivity. In addition to reducing greenhouse gas emissions, microalgal cultivation generates oxygen and produces commercially valuable products such as pigments, antioxidants, proteins, and lipids, thereby improving the overall sustainability of the process. Fig. 5 illustrates the integrated microalgal carbon capture pathway, demonstrating the conversion of CO_2_ into biomass and various value-added products through biological fixation.^1,37^

**Fig. 5:**
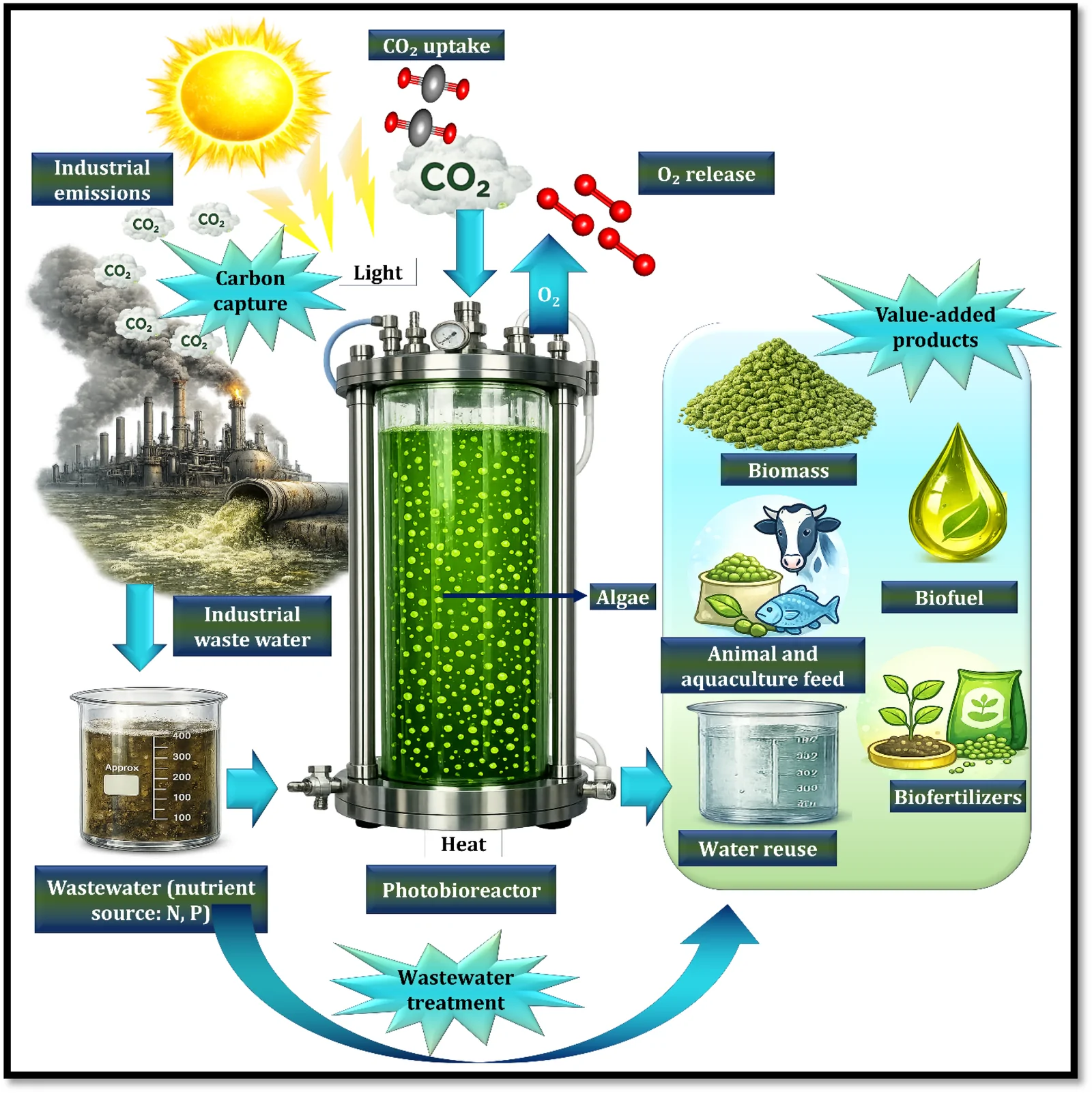
Microalgae-based carbon capture and conversion pathways for biomass and value-added product generation. Adapted from ref. 37, published by Frontiers under the creative commons attribution (CC BY) License. Copyright © 2020.

Despite these environmental advantages, several technical and economic challenges limit large-scale commercialization of microalgal CO_2_ capture. Large cultivation areas, high water demand, energy-intensive harvesting and dewatering, and costly downstream processing substantially increase operating costs, while biomass productivity is sensitive to climatic conditions, seasonal variability, and culture contamination. As a result, the production of low-value products such as biofuels is often economically unfavorable, and integrated biorefinery concepts that recover multiple high-value compounds from algal biomass are increasingly explored to improve process economics and resource utilization.^37^

From a critical perspective, microalgal CO_2_ capture provides the unique advantage of directly converting carbon emissions into renewable biomass while contributing to a circular carbon economy. However, unlike absorption-based technologies that have already achieved commercial deployment across numerous industrial sectors, most microalgal systems remain at the laboratory or pilot scale. Their performance varies considerably depending on algal species, cultivation conditions, and reactor design, making direct comparison among reported studies difficult. Moreover, the scalability and economic viability of large-scale microalgal cultivation remain uncertain without significant improvements in cultivation efficiency, biomass harvesting, and downstream processing. Therefore, although biological carbon capture represents a promising complementary strategy for long-term carbon utilization, absorption-based CO_2_ capture continues to be the most technologically mature and economically feasible option for immediate industrial implementation. For this reason, the subsequent sections focus primarily on absorption technologies, solvent development, desorption enhancement strategies, and techno-economic feasibility, which constitute the principal scope of this review.

### 2.4 Adsorption-based technologies for carbon dioxide capture

The CO_2_ adsorptive capture is a well-known and effective approach to capture and condense carbon dioxide to mixtures of gases, including flue gases and atmospheric air.^10^ The method involves the use of porous solid materials which have a high physicochemical affinity to CO_2_ molecules. When the gas stream is in touch with these adsorbents, CO_2_ is absorbed selectively onto the surface of the material, leaving the other gases passing through it and consequently, efficient separation can be achieved. An average setup of the adsorption system is shown in Fig. 6.^39^ The choice of the adsorbent has a critical impact on the success of this method since the structural and surface properties of the adsorbent predetermine the overall capture efficiency.^38^ Activated carbon, zeolites, and metal–organic frameworks (MOFs) are the most commonly used adsorbents that vary in terms of pore structure, surface activity, and adsorption capacity and can be fine-tuned to fit certain applications.^38^ Once the adsorbent has been saturated, it becomes possible to regenerate it by changing operating conditions typically using temperature swing adsorption (TSA), pressure swing adsorption (PSA), or a combination of both methods and release the trapped CO_2_ to be reused. Essentially, the adsorption-based CO_2_ capture provides a very versatile and comparatively low-energy solution to emission control. Its flexibility in operations and applicability to different industrial systems render it a very attractive choice in the development of carbon capture and utilization technologies.

**Fig. 6:**
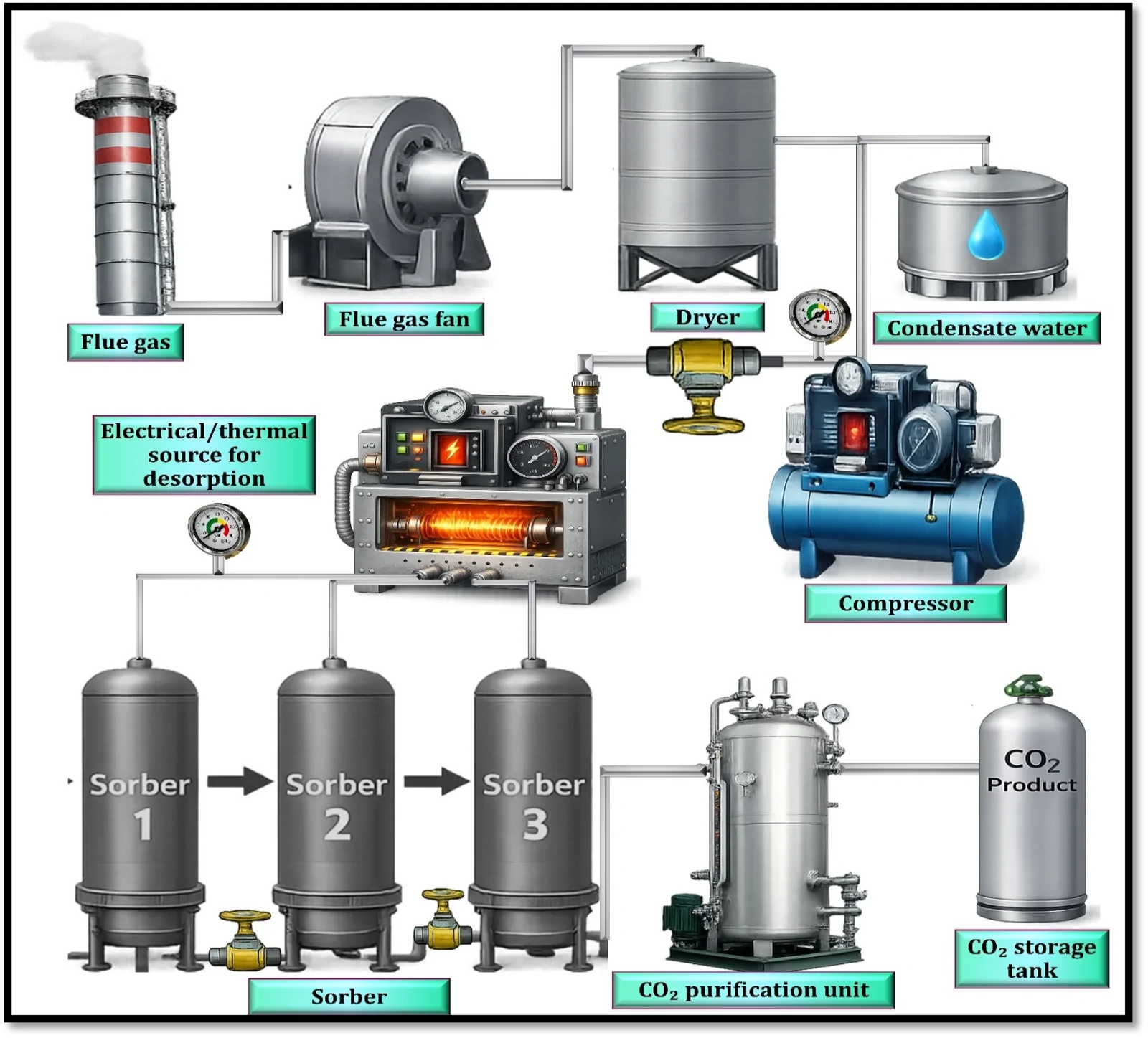
Schematic representation of a conventional adsorption process for gas separation. Adapted from ref. 39, licensed under a CC BY-NC-ND 4.0.

The energy requirement of adsorption-based CO_2_ capture is relatively low because the interactions between CO_2_ molecules and the adsorbent surface are predominantly weak, non-covalent interactions rather than strong chemical bonds. This lowers the energy needed for regeneration and can improve overall efficiency and cost-effectiveness. Adsorption performance arises from multiple interacting mechanisms, including heterogeneous surface interactions, electrostatic forces, acid–base affinities, physical adsorption, diffusion through porous structures, molecular sieving, and hydrogen bonding. Well-defined narrow pore networks, particularly those modified with CO_2_-attractive heteroatoms, are especially beneficial for enhancing selectivity and uptake. In addition, practical adsorbents must exhibit high mechanical robustness, high CO_2_ selectivity in mixed gas streams, thermal stability, low-cost synthesis, and tolerance to humid conditions to ensure consistent performance in real industrial environments. However, despite these advantages, large-scale deployment of adsorption-based capture is still constrained by adsorbent cost, sensitivity to impurities and moisture, and the need for complex TSA/PSA regeneration cycles, which often makes adsorption more suitable for specific niche applications rather than widespread replacement of absorption-based systems.

Table 3 indicates that conventional amine solvents such as MEA, DEA, and MDEA still form the backbone of industrial absorption-based CO_2_ capture, but each occupies a distinct performance niche. MEA offers high capture efficiency and a well-established process design, yet suffers from high regeneration energy demand and corrosion, making it costly to operate at scale. DEA and MDEA reduce corrosion or regeneration energy penalties, but trade this off against slower kinetics and the need for higher contact times, which complicates column design and increases equipment size. Newer systems—including piperazine-promoted amines, sterically hindered amines, promoted carbonate solutions, chilled or aqueous ammonia, ionic liquids, and AI-designed solvents—aim to improve cyclic loading and lower regeneration energy, but frequently introduce new challenges such as precipitation, solvent loss, high viscosity, or high material costs. Overall, the comparison in Table 3 shows that solvent innovation has shifted the performance frontier rather than eliminating core trade-offs between energy demand, stability, and cost, reinforcing the need for a unified solvent comparison and techno-economic framework that explicitly couples capture capacity and regeneration penalties to long-term operating expenditure.^32^

**Table 3:** Comprehensive review of absorption-based CO_2_ separation technologies

S.No	Absorption technology/solvent system	Absorbent type	Working principle	CO_2_ capture capacity/performance	Advantages	Limitations	Typical industrial application	Ref.
1	Monoethanolamine (MEA)	Primary amine	85–90% Efficiency	High reactivity, proven technology	High energy demand, corrosion	Power plant post-combustion capture	Post-combustion CO_2_ capture in coal- and gas-fired power plants	39
2	Diethanolamine (DEA)	Secondary amine	Moderate absorption	Lower corrosion	Slow kinetics	Natural gas sweetening	Natural gas sweetening and acid gas removal	40
3	Methyldiethanolamine (MDEA)	Tertiary amine	High CO_2_ selectivity	Low regeneration energy	Slow reaction rate	Gas processing	High-pressure natural gas processing and refinery gas treatment	41
4	Piperazine-promoted amines	Amine blend	Faster than MEA	High cyclic capacity	Precipitation issues	Advanced PCC systems	Advanced post-combustion CO_2_ capture systems	42
5	Hot potassium carbonate (HPC)	Carbonate solvent	Good for high-pressure CO_2_	Low toxicity, low cost	Slow kinetics	Syngas purification	Pre-combustion capture and syngas purification	43
6	Chilled ammonia process	Aqueous ammonia	High CO_2_ loading	Lower regeneration energy	Ammonia slip	Coal-fired plants	Coal-fired power plant flue gas treatment	44
7	Aqueous ammonia absorption	Inorganic solvent	High absorption capacity	Low solvent cost	Ammonia loss	Flue gas capture	Industrial flue gas CO_2_ capture	45
8	Ionic liquid absorption	Ionic liquids	Tunable CO_2_ solubility	Thermal stability	High cost, viscosity	Advanced CCS	Advanced carbon capture systems	46
9	Sterically hindered amines	Modified amines	Higher cyclic loading	Lower regeneration energy	Slow kinetics	Advanced absorption units	Advanced absorption processes	47
10	Promoted potassium carbonate	Carbonate + activator	Improved absorption rate	Low corrosion	Requires promoters	IGCC, syngas treatment	IGCC and syngas treatment	48
11	AI-designed amine solvents	Optimized amines	Higher predicted efficiency	Rapid solvent discovery	Experimental stage	Future CCUS technologies	Next-generation CCUS technologies	48

While membrane, cryogenic, microalgal, and adsorption-based technologies each offer distinct advantages for specific applications, their large-scale deployment remains constrained by challenges including high capital cost, energy demand, membrane fouling, limited scalability, or operational complexity. In contrast, absorption-based CO_2_ capture remains the most technologically mature and commercially implemented approach, combining high capture efficiency and operational flexibility with established industrial infrastructure. Therefore, the remainder of this review primarily focuses on recent advances in absorption processes, solvent development, desorption enhancement strategies, and techno-economic evaluation to provide a more comprehensive and in-depth critical assessment consistent with the scope of this review.

### 2.5 Carbon dioxide capture through absorption processes

Carbon dioxide (CO_2_) capture *via* absorption is an established method of capturing gaseous emissions especially the emissions of power generation and industrial processes. The process works on the principle of selective removal of CO_2_ in a gaseous state to a liquid media that has a high affinity to the gas. In practice the separation is achieved in a contact device, often an absorption column, to ensure the maximum contact between the incoming gas stream and the liquid solvent. Since the gas is rising up the column as the solvent is moving in the opposite direction, the concentration gradients cause diffusion of the CO_2_ molecules into the liquid and the physicochemical interactions are displayed in Fig. 7. This leads to the exit of a CO_2_ depleted gas stream out of the system and the enriched solution of CO_2_ is obtained. The solvent with CO_2_ is then pumped to a regeneration unit where thermal energy is used to undo the absorption process.^49^ High temperatures dilute the interactions between CO_2_ and solvent leading to the release of concentrated CO_2_ gas. Meanwhile, the solvent is returned to its working stage and can be recycled to the absorber to reuse again, improving the efficiency of the process and its economic feasibility. The retrieved CO_2_ can subsequently be subjected to compression and channeled to the storage or utilization routes.^50^ These can be either subsurface sequestration, enhanced oil recovery, or the conversion of these products to commercially viable products. Due to the operational maturity, scalability and effectiveness, absorption is one of the cornerstone technologies in carbon capture systems.

**Fig. 7:**
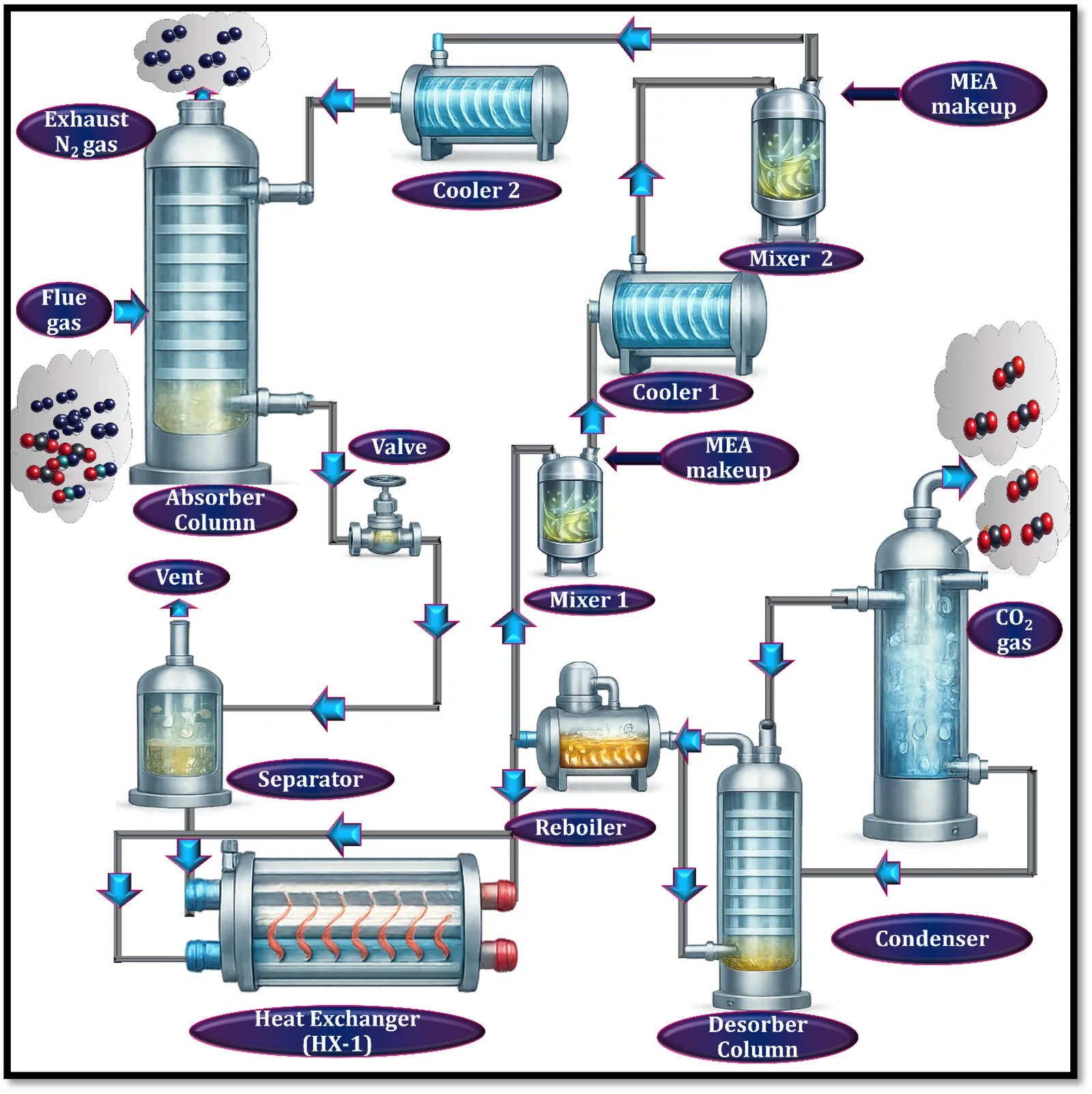
Conceptual illustration of CO_2_ capture *via* conventional chemical absorption techniques. Adapted from ref. 39, with permission from Elsevier. Copyright © Elsevier. License number: 6298771290399.

The reason behind the popularity of absorption technology is that it is considered to be one of the most successful methods of capturing CO_2_ on an advanced level due to its higher selectivity and high level of uptake. The properties permit efficient separation of both carbon dioxide and complex gas streams and the process is particularly attractive to large-scale users, such as in power generation and industrial pollution removal. However, this widespread applicability often masks significant thermodynamic inefficiencies that become more pronounced at industrial scale, particularly when integrated into existing power systems. However, it is limited in its practical implementation by a number of its inbuilt weaknesses. The high energy requirements of the solvent regeneration that comes with the process is a key issue as it has a significant effect on the overall efficiency of the process. In fact, regeneration energy can constitute a major fraction of total process energy demand, thereby reducing net CO_2_ mitigation benefits. Moreover, its use is also complicated by operational challenges like corrosion of equipment, environmental and health issues caused by solvent toxicity and high costs of absorbent material. Absorbent stability is also a concern since, over time, performance may decline due to degradation and evaporation losses, and may require more maintenance.^51^ These degradation pathways are often underestimated in laboratory studies, leading to discrepancies between pilot-scale performance and real industrial operation. These constraints combined are serious impediments to extensive implementation of absorption-based CO_2_ capture systems. Table 4 gives a detailed summary of different absorbents and their effectiveness.^52–58^

**Table 4:** Comparative performance of representative absorption solvents for CO_2_ capture under different operating conditions

S.No	Absorbents	Concentration	Diffusion coefficient *D*(CO_2_) (m^2^ s^−1^)	CO_2_ capture capacity	Temperature (°C/K)	Adsorption equilibrium (min)	Ref.
1	Monoethanolamine (MEA)	30 wt%	1.8 × 10^−9^	0.40–0.50 mol CO_2_/mol amine	40–60 °C (313–333 K)	20–40	52
2	Diethanolamine (DEA)	20–30 wt%	1.5 × 10^−9^	0.35–0.45 mol CO_2_/mol amine	40–70 °C (313–343 K)	30–50	53
3	Methyldiethanolamine (MDEA)	40–50 wt%	1.2 × 10^−9^	0.50–0.70 mol CO_2_/mol amine	40–80 °C (313–353 K)	40–70	54
4	Piperazine (PZ) activated MDEA	5 wt% PZ + 40 wt% MDEA	1.9 × 10^−9^	0.75–0.85 mol CO_2_/mol solvent	40–60 °C (313–333 K)	15–30	55
5	Potassium carbonate (K_2_CO_3_)	20–30 wt%	0.9 × 10^−9^	0.30–0.50 mol CO_2_/mol solvent	50–75 °C (323–348 K)	50–90	55
6	K_2_CO_3_ + MEA promoted solution	30 wt% K_2_CO_3_ + 5–10 wt% MEA	1.4 × 10^−9^	0.65–0.80 mol CO_2_/mol solvent	63 °C (336 K)	20–45	55
7	K_2_CO_3_ + TETA	1.5 M K_2_CO_3_ + 1 M TETA	1.6 × 10^−9^	0.65–0.80 mol CO_2_/mol solvent	303–323 K	25–60	56
8	Aqueous ammonia (NH_3_)	5–15 wt%	2.0 × 10^−9^	0.90–1.20 kg CO_2_/kg solvent	20–40 °C (293–313 K)	10–25	57
9	Chilled ammonia process	2–10 wt% NH_3_	2.2 × 10^−9^	0.95–1.25 kg CO_2_/kg solvent	0–10 °C (273–283 K)	10–20	57
10	Amino acid salt solutions (glycine/sarcosine)	1–3 M	1.3 × 10^−9^	0.60–0.85 mol CO_2_/mol solvent	25–60 °C (298–333 K)	20–50	57
11	Piperazine (PZ)	8 m PZ	2.0 × 10^−9^	0.80–1.00 mol CO_2_/mol solvent	40–70 °C (313–343 K)	10–25	58
12	AMP (2-amino-2-methyl-1-propanol)	20–30 wt%	1.4 × 10^−9^	0.50–0.65 mol CO_2_/mol amine	40–70 °C (313–343 K)	30–60	58

Table 4 shows that solvents with higher CO_2_ capacities and faster kinetics (*e.g.*, promoted amine and ammonia-based systems) can reduce contact times and potential regeneration energy, but they also introduce added challenges in solvent stability, management, and process complexity. This highlights that solvent selection cannot rely on capacity alone; it must balance uptake, diffusion, kinetics, operating conditions, and long-term stability, which is why this review develops a unified solvent comparison and techno-economic framework instead of treating each property in isolation.

Although amine-based solvents such as MEA remain the industrial benchmark because of their high CO_2_ absorption rate and technological maturity, they are associated with high regeneration energy requirements, solvent degradation, and equipment corrosion. This indicates a fundamental trade-off between reactivity and energy efficiency that has not yet been fully resolved. Blended amines, promoted carbonate solutions, amino acid salts, and ammonia-based solvents have been developed to address these limitations by reducing regeneration energy, improving cyclic stability, or enhancing absorption capacity. However, these alternatives often introduce new challenges such as slower kinetics, higher viscosity, or increased process complexity. Nevertheless, most emerging solvent systems have been evaluated primarily under laboratory or pilot-scale conditions, and their long-term operational stability, solvent management, and economic feasibility under industrial operating conditions require further validation. This gap highlights a critical limitation in current research, where scalability and lifecycle performance are not sufficiently addressed. Consequently, no single absorbent currently satisfies all technical, economic, and environmental requirements, highlighting the need for continued solvent optimization and process integration.

The absorption process of carbon dioxide can be broadly classified based on the two distinct processes: physical and chemical absorption, which are differentiated by the way of interaction between CO_2_ and the solvent.^59^ In physical absorption, CO_2_ is dissolved in a liquid state without any chemical change taking place and its solubility is regulated by Henry law that relies on the solubility of gases as the product of gas partial pressure and solubility. While this simplifies regeneration, it inherently limits effectiveness at low CO_2_ partial pressures, restricting its applicability in post-combustion systems. Chemical absorption, on the other hand, is characterized by reactive solvents, which react with CO_2_ to create reversible compounds to capture selectively an even lower concentration. Under high pressure, low temperature conditions, which maximize the CO_2_ solubility of the solvent are preferred, physical absorption processes are employed. The solvent regeneration here is not as complex and can be achieved by reducing the pressure of the system, raising the temperature or a combination of both. Despite this advantage, the requirement for gas compression introduces a significant indirect energy penalty that can offset the benefits of easier regeneration. Although this advantage exists, one of the significant limitations is that a lot of energy is needed to compress the flue gases to the high pressure needed to optimally absorb.^60^

The principle of chemical absorption on the other hand relies on the formation of weakly bound intermediate species between solvent and CO_2_. These intermediates are also able to be broken down when heated, giving up the CO_2_ absorbed, and the solvent is reused. This process works best in the case of low CO_2_ partial pressures of gas streams and consequently it works best in post combustion capture. The most common solvents used include the amine based solutions and alkaline carbonate systems among others of which the amine based solutions and alkaline carbonate systems are highly reactive and capture efficient. However, this high reactivity is directly linked to higher regeneration energy and increased solvent degradation, reinforcing the inherent trade-off in chemical absorption systems. The CO_2_ in physical absorption schemes is absorbed in the bulk of the solvent and, consequently, the technique is especially effective at high pressure, such as pre-combustion capture in integrated gasification combined cycle (IGCC) systems. The process of flash desorption or stripping is used to regenerate the CO_2_ contaminated solvent after absorption. Flash desorption is a desorption which makes use of phases of decreasing pressure which allows the dissolved CO_2_ to evaporate out of the solvent. Alternatively, a combination of pre-depressurization and inert gas injection (typically nitrogen) is attained by stripping in order to remove residual CO_2_. These regeneration measures guarantee the restoration of CO_2_ with the solvent remaining effective after several working cycles.^61^ In practice, however, repeated cycling often leads to solvent fatigue, accumulation of impurities, and reduced absorption efficiency, necessitating additional purification or replacement strategies that increase operational costs.

## 3 Emerging innovations in absorptive CO_2_ capture processes

Carbon dioxide (CO_2_) capture based on absorption has significantly developed in recent years making it one of the technologies of efficient gas separation. The technique has been extensively studied with a variety of different solvent systems that can be categorized into physical, chemical and blended systems depending on their mechanism of interaction with CO_2_. CO_2_ is dissolved by the physical solvents by dissolution dominated by weak intermolecular forces without creating any chemical bond. Solvents such as Rectisol, propylene carbonate and Selexol among others can be used and are most effective in high-pressure and low temperature environments where the CO_2_ solubility is significantly enhanced. On the other hand, chemical solvents like monoethanolamide (MEA), diethanolamine (DEA) and methyl diethanolamine (MDEA) undergo reversible chemical reactions with CO_2_. This mechanism is very useful in increasing the selectivity and absorption efficiency particularly in gas streams that contain low concentrations of CO_2_. Their high reactivity and an established industrial application make them key to numerous existing capture systems. Absorption-based CO_2_ capture performance is very sensitive to the solvent used. Absorption efficiency, regeneration energy requirements, degradation resistance, cost, and corrosiveness, are the most important considerations. As a result, the identification and optimization of suitable solvents continue to be at the core of enhancing the feasibility and scaleability of this technology.^62^

Notably, the chemical absorption systems are well valued due to their strength in terms of administration of acid gas removal with a wide range of operating conditions. The significant advantage is that they are not highly dependent on the partial pressure of the gases which are acidic and thus can be employed in capturing the minute amount even at parts-per-million levels. The solvents also support a fast rate of the transfer of mass on both the uptake and release processes, which leads to a high separation efficiency.^62^ However, these benefits are offset by a number of operational and economic issues. The process of the regeneration is very energy demanding since the solvent and absorbed gases can react with each other intensively, and thus, the temperature of absorption is very high. Additionally, chemical solvents are not always highly selective in the presence of many acid gases and tend to exhibit corrosion of equipment, solvent degradation through side reactions and other environmental hazards. Another issue that can arise in aqueous systems is that the treated gas is likely to saturate with water vapor, and probably needs additional processing. Conversely, physical solvents are dissolved by non-covalent dissolution processes and do not form any covalent bonds to the dissolving agent and thus, the amount of energy needed to regenerate the solvent is considerably low. The reduced thermal demand coupled with relatively small heats of absorption renders them desirable in some applications. They are also capable of providing better selectivity in the favorable conditions.^63^ Their performance is however closely related to the partial pressure of acid gases which restricts their performance in the dilute and low-pressure streams. Under these circumstances, they are likely to have a lower CO_2_ capture capacity, and the absorption and desorption rates are usually lower than those of chemical systems. Moreover, there are still physical solvents that can pose practical difficulties such as increased costs of procurement, corrosion-related problems and environmental effects that can be caused by the solvent contents.^64^

Blended solvent systems have proven to be an effective method in capture of CO_2_ by absorption, with the aim to utilise the complementary properties of various solvents in a single formulation. Integrating several elements, these mixtures may provide a better CO_2_ capture, a higher selectivity in the presence of other gases, and lower energy needs during the regeneration process. Such systems tend to either be hybrid mixtures of physical and chemical solvents or highly-designed mixtures of chemical absorbents that are synergistic.^62^ Much research has been focused on the identification of high-performance solvent blends by screening and optimization. Here, Nwaoha *et al.* studied concentrated ternary mixtures of 2-amino-2-methyl-1-propanol (AMP), piperazine (PZ) and monoethanolamine (MEA) in the context of capturing CO_2_. They found that these blends are more effective than conventional 5 kmol m^−3^ MEA solutions in several important aspects, including the higher capacity of the cycles and quicker initial desorption kinetics. Significant reductions in energy requirement of regeneration of 50–54.5% were observed also that indicates substantial increases in the efficiency of the processes.^65^ Besides, AMP to PZ solvent compositions, AMP : PZ = 1 : 2 showed higher initial absorption rates and this supports the importance of compositional tuning. Although the reported reduction in regeneration energy (50–54.5%) demonstrates the potential of AMP–PZ–MEA blended solvents, these results were primarily obtained under controlled laboratory conditions and may not be directly transferable to industrial applications. Their practical implementation requires further validation under varying flue gas compositions, long-term cyclic operation, and pilot-scale studies to assess solvent stability, degradation, and economic feasibility. Furthermore, while several studies consistently report improved absorption kinetics for blended solvents, differences in solvent composition, operating conditions, and reactor configurations make direct comparison difficult, highlighting the need for standardized evaluation protocols.

An experiment was conducted in depth to determine the solubility and the mass transfer properties of CO_2_ in unmodified and promoted solutions of a high temperature potassium carbonate (K_2_CO_3_). The thermodynamic model was considered to be effective since the experimental results at 80 °C, 100 °C and 120 °C with 1.81 M and 2.53 M solvent concentrations showed that the electrolyte-NRTL model strongly matched the experimental findings. To enhance the performance, solutions of 1.81 M K_2_CO_3_ were introduced with various promoters such as PZ, MEA and glycine and run at 100–120 °C. CO_2_ uptake and absorption kinetics increased significantly in PZ and MEA which implied that they could be used as promoters. Glycine only increased the rate of absorption at lower CO_2_ loadings but the overall capacity was not changed significantly. On the other hand, MDEA demonstrated a positive reaction in higher concentrations, decreasing the solvent ability and failed to enhance the rate of absorption.^66^ Along with carbonate-based systems, ionic liquid (IL)-based solvent platforms have undergone study with an aim of overcoming the drawbacks of standard amine solvents. Such benefits as the possibility of a zero vapour pressure and high thermal stability are associated with ionic liquids, such as [Bmim][BF4]. and [Bmim][PF6]. The typical amines, including MEA and MDEA, are more likely to provide increased CO_2_ loading, but hybrid IL-amine systems have been observed to provide enhanced overall performance due to the combination of the chemical reactivity of amines and physical stability of ILs. Specifically, the [Bmim][BF_4_] mixtures with MEA had stronger CO_2_ absorption than pure ILs or aqueous amines and the best composition of the mixture was found to be 80% IL and 20% MEA. CO_2_ uptake was further enhanced by the addition of a second promoter, which was piperazine. Comprehensive kinetic analysis of such blends gave an understanding of their improved reaction and transport properties.

To address the high energy cost in solvent regeneration of aqueous amine systems, novel water-lean biphase amino acid salt-based absorbents have been suggested. Systems that have potassium prolinate (ProK) and potassium sarcosinate (SarK) and 2-alkoxyethanols as phase-modifying agents show unusual phase separation behavior during CO_2_ absorption. Weak polar solvents (2-methoxyethanol and 2-ethoxyethanol) allow the formation of solid phase and facilitate successful CO_2_ capture in the form of ionic species. About 50–80% of the CO_2_ absorbed in such systems remains in the solid slurry phase. A 3.0 M ProK/EGME system had a similar cyclic capacity and much better desorption efficiency and regeneration energy requirements, 40–50 times lower than the traditional 5.0 M MEA systems. This demonstrates that biphasic amino acid-based systems have a high potential as energy efficient alternative energy sources when used in the advanced CO_2_ capture application.^67^

There is increasing interest in the use of deep eutectic solvents (DESs) as a better medium in the capture of carbon dioxide because of their structural flexibility and ability to regulate intermolecular interactions. Recently, a series of DES formulations have been developed, which were composed of monoethanolamide hydrochloride (EAHC) with a range of amino-based hydrogen bond donors, such as ethylenediamine (EDA), monoethanolamine (MEA), diethylenetriamine (DETA), triethylenetetramine (TETA), and tetra as it was observed, CO_2_ solubility was most in the DESs which contained more amine component (that is 1EAHC : 9DETA, 1EAHC : 9TETA and 1EAHC : 9TEPA). It is linked with this enhancement because a greater concentration of reactive amine groups and because of the influence of long alkyl chains that increase the interaction with the CO_2_ molecules and result to the increased absorption.^68^ These results suggest that such DES systems could be applied to compete with the conventional solvents in the gas capture process.

Along with solvent composition, surfactant incorporation in the adaptation of the absorption medium has also been an effective modification to increase the CO_2_ capturing capacity. In particular, it has been determined that adding frothing agents to the sodium carbonate (Na_2_CO_3_) solutions can greatly increase the absorption efficiency to 99.9% when compared to absorption efficiency of 55.6% when carbonate is not added. This dramatic increase can be attributed to a great extent to increased gas–liquid contact properties. Surfactants reduce the surface tension, allowing smaller and more uniformly distributed bubbles to form and reducing their tendency to fuse. This causes the interfacial area to increase significantly, and facilitates greater mass transfer, thus increasing the overall CO_2_ absorption rate.^69^

The particular interest in recent years has been a special category of absorbents called phase-change solvents, due to their ability to significantly reduce the energy requirements of CO_2_ capture. Under usual conditions, these solvents are single-phase systems. Nevertheless, the system experiences a transformation to two distinct phases when important properties like polarity, hydrophilicity, ionic strength or hydrogen-bonding interactions are modified. This induced phase separation creates a CO_2_ lean phase and the other phase which is highly enriched with CO_2_ and can be either liquid or solid. This type of behavior allows selective concentration of CO_2_ and enhances the efficiency of the process in the regeneration process.^70^ Very many different types of phase-change solvent systems have been studied, some of which are responsive to thermal or chemical stimuli. These systems either can be aqueous or non-aqueous, and their phase shift can lead to the appearance of a layer with a high concentration of CO_2_ or the formation of a solid phase, depending on the composition of the solvent and the conditions of operation.^71^

There has been an increasing literature on the use of phase change solvents, both aqueous and non-aqueous, to capture carbon dioxide. In non-aqueous systems, water is replaced by organic diluents or ionic liquids and in so doing, solvent regeneration can be achieved at comparatively lower temperatures. This solution overcomes various intrinsic drawbacks of aqueous amine-based systems such as thermal and oxidative destruction, solvent volatility, equipment corrosion, and the high energy requirements of regeneration.^72^ Nevertheless, the non-aqueous amine solvents practical implementation on an industrial level is limited by such issues as the fact that their viscosity is high and that the species created are not readily soluble. These problems may affect the ability of fluid flow and cause fouling or clogging of processing units. To address these limitations, more recent studies have suggested an alternative solvent system in which 1,5-diamino-2-methylpentane (DA2MP) is the main CO_2_-reactive compound with 2-amino-2-methyl-1-propanol (AMP) being used as a functional additive to control viscosity and dissolve precipitated species. The resulting solvent formulation was found to have high CO_2_ loading capacity of 0.95 mol per mol of absorbent and it also experienced a significant reduction in viscosity as compared to the conventional systems. It was also characterized by a high level of cyclic stability as it was able to retain approximately 97% of its initial absorption capacity following four regeneration cycles.^73^ This is because these are credited to the changes in the hydrogen-bonding network in the solvent which allows reduced intermolecular resistance and prevents the formation of insoluble products. Moreover, the amount of energy needed to regenerate the solvents was reduced significantly by 50.27 per cent of the amount needed to regenerate the solvents using monoethanolamine (MEA) processes. Taken together, these characteristics make the proposed solvent system a very promising candidate to creating an efficient and sustainable CO_2_ capture.

Various phase-change solvent-based methods of CO_2_ capture have been proposed, each with the objective of capitalizing on phase separation to maximize absorption capacity, and minimise regeneration energy usage. Some of them include the self-concentration process, the iCap technology, the DMX system, the TBS approach, and the DECAB method.^70^ The process of self-concentration makes use of a non-aqueous formulation of a mixture of amine and alcohol. The initially homogeneous solution spontaneously separates into two phases of liquids, under absorption conditions, usually at temperatures near 35 °C. The enriched fraction that contains CO_2_, which is known as the rich phase, is heavier and is separated by decanter. This stage is fed only to a stripping unit, where it is regenerated at temperatures between 115 °C and 125 °C. After the CO_2_ is removed, the regenerated solvent is recirculated into the absorption column back to a continuous capture cycle. Conversely, iCap process is an aqueous solvent mixture of MAPA (2 M) and DEEA (5 M). This system also shows the liquid–liquid phase separation during CO_2_ uptake. Nevertheless, it is only the CO_2_ rich fraction of the solvent that is channelled to the regeneration process with the lean phase directly recirculated to the absorber. The focused regeneration scheme also minimizes the quantity of thermal energy utilized in that the quantity of solvent that is warmed is minimized.^74^ Similarly, other mechanisms such as DMX, TBS and DECAB are also premised on the same principle of separating the phases during the process of absorbing CO_2_. By focusing the captured CO_2_ at a specified phase, these systems can selectively regenerate the CO_2_, resulting in energy penalties that are reduced and overall process performance is increased.

Amino acids ternary system of solvents which include amino acids, amines too are under consideration to be used as effective substitutes to capture CO_2_ as these have the ability to blend the functionalities of different alkanolamines to a single effective formula. These blends when appropriately utilized have cooperative effects that in most instances drastically enhance absorption performance, even greater than standard aqueous monoethanolamine (MEA) when the same is used in the same scenario.^75^ Research has shown that aqueous solutions that contain a primary, secondary amine with a tertiary or sterically hindered amine react rapidly in comparison to systems which utilise tertiary or sterically hindered amines. Besides enhanced kinetics, these mixed solvents usually require less energy in regeneration as compared to single-component primary or secondary solutions made of amines.^76^ The improved behavior of these systems is explained by the complementary, but different roles of the components. Primary or secondary amines improve fast absorption of CO_2_ and transfer mass properties whereas tertiary or sterically hindered amines are more of proton acceptors and this promotes efficient recovery of solvents. Such separation of roles allows ternary amine blends to provide a high absorption capacity and lower regeneration energy and thus are good candidates in high-technology carbon capture.^75^

### 3.1 Advanced strategies for enhancing CO_2_ desorption efficiency

Enhancing the CO_2_ desorption is a key to the development of the carbon capture and storage (CCS) systems because the regeneration is the stage that defines the energy consumption, as well as the efficiency of the process, to a large extent. The objective of improving desorption is to ensure that the release of CO_2_ by absorbents following capture is faster and more efficient. This is intrinsically challenging since CO_2_ is a very recalcitrant molecule, its carbonoxygen double bonds are robust and it is not reactive, and thus the separation of the compound with solvents is thermodynamically challenging. As such, much research has been done on the development of strategies to enable the release of CO_2_ at lower energy penalties of bulk absorbent phases. A number of improvement methods have been studied to overcome such difficulties such as the nanofluids, solid acid catalysts, enzymatic or biocatalytic systems, and ionic liquids. All these methods are aimed at enhancing mass transfer, reaction kinetics, or heat transfer in the regeneration process. Nanofluids have become one of them especially because they are capable of dramatically changing the nature of the physicochemical properties of traditional solvents.^77^

Nanofluids are designed by dispersing the nanoscale particles in a base liquid, which can be water, alcohols or organic solvents. These particles can be made up of metals, metal oxides, carbides or carbon based nanostructures. Their introduction improves thermal conductivity, interfacial area and micro-level mixing in favor of desorption behavior. The nature of the nanoparticles used greatly influences the effectiveness of nanofluids. It has been demonstrated through experimental research that the addition of silica (SiO_2_) nanoparticles at the right concentration can increase the CO_2_ regeneration capacity by almost 12%, mainly because of the enhanced heat and mass transfer properties. Alternatively, alumina (Al_2_O_3_) nanoparticles have been found to have adverse effects on performance and lead to up to about 15 reductions. This negative outcome is explained by the fact that the condition of chemical interaction between CO_2_ and Al_2_O_3_ surface results in the formation of stable bicarbonate and carbonate species that hinder the release of CO_2_. This kind of interaction is of no consequence in the SiO_2_ case, and that is why it is a more appropriate additive to be used in enhancing desorption.^78^ Moreover, it has also been determined that incorporation of nanoparticles enhance absorption as well as desorption in physical solvents like methanol, meaning that they are more applicable in optimization of CO_2_ capture systems.

Hafizi *et al.* examined the regeneration performance of nanofluids with amine-functionalized Fe_3_O_4_ nanoparticles by assessing traditional thermal regeneration and another method ultrasonic bath. Their results proved that it was possible to use ultrasonic treatment to cause a very fast CO_2_ release and reach a desorption in less than a minute and maintain the efficiency of the absorbent in repeated cycles.^79^ This implies that the ultrasound-based regeneration provides a very efficient and time-saving process compared to traditional heating-based processes.

Simultaneously, solid acid catalysts have been considered as efficient catalysts of CO_2_ desorption due to their intense surface activity, reusability, and stability under the current conditions. These catalysts enable the release of CO_2_ at low temperatures relative to non-catalytic processes, hence consuming much less thermal energy. Moreover, they help to enhance the CO_2_ release and facilitate a simple separation of the catalyst and liquid phase.^80^ This results in the regenerated absorbent maintaining the original capture properties, and can be used to counter the traditional trade-off between large absorption capacity and the energy-consumptive regeneration.

Recently, there have been studies that aim at integrating solid acid catalysts with membrane-based apparatus to enhance the rate of CO_2_ desorption of aqueous monoethanolamine (MEA) solutions. One study incorporated H-ZSM-5 into an α-Al_2_O_3_ ceramic membrane, and the performance of HZSM-5 was systematically tested under various operating conditions to learn how it influences CO_2_ release and energy consumption.^81^ This system was shown to have a high desorption efficiency, and a stable behavior in 60 consecutive cycles, which validated its stability. Such findings suggest that a strong acid catalyst in combination with a porous ceramic support can greatly enhance the desorption processes and reduce the energy required.

Likewise, Bhatti *et al.* investigated the use of mesoporous HZSM-5 catalysts to overcome the natural drawback of traditional MEA regeneration, which frequently requires the use of high thermal energy and is slow in desorption kinetics. The catalysts designed, with increased surface area and well developed mesoporosity, significantly improved the desorption performance.^80–86^ A remarkable increase in desorption rate up to 580% at 82 °C was observed, along with a 60% improvement in the total amount of CO_2_ released. Moreover, the energy need to regenerate was decreased by 37%. The outcomes demonstrate that the structural properties of catalysts play a vital role in enhancing CO_2_ capture efficiencies and provide valuable insights on how to optimize the design of advanced catalytic systems. Previous research on CO_2_ enhancement methods is provided in Table 5.

**Table 5:** Evaluation of solid catalytic materials in CO_2_ regeneration systems

S.No	Catalyst	Conditions	Main outcome	Ref.
1	Mesoporous HZSM-5	30 wt% MEA, 40–82 °C	↑ Desorption rate (350–580%), ↓ heat duty (24–37%)	80
2	HZSM-5	25 wt% amine, ∼80 °C	Improved desorption (depends on loading & temp)	81
3	SnO_2_/ATP	5 M MEA, 88 °C	↑ Rate (265%), ↓ energy (27%)	82
4	Fe–SO_4_^2−^/ZrO_2_ (SZM)	MEA, 98 °C	↓ Heat duty (∼20–38%)	83
5	Ni–HZSM-5	90 °C	↓ Heat duty (∼27%), stable	84
6	SO_4_^2−^/ZrO_2_–HZSM-5	<98 °C	↓ Energy (∼31%)	85
7	Composite (CeO_2_, HPW, *etc.*)	<98 °C	↓ Energy (16–29%)	86

Overall, recent studies consistently demonstrate that advanced solvent formulations can significantly improve CO_2_ capture performance and reduce regeneration energy requirements. However, reported improvements often originate from laboratory-scale investigations performed under different operating conditions, solvent concentrations, and gas compositions, making direct comparison challenging. Future research should prioritize standardized testing protocols, long-term stability assessments, pilot-scale demonstrations, and comprehensive techno-economic analyses to establish the industrial viability of these emerging absorption technologies.

### 3.2 Machine learning and artificial intelligence-assisted solvent screening

Recent advances in machine learning (ML) and artificial intelligence (AI) have significantly accelerated the discovery and optimization of absorbents for CO_2_ capture.^87^ Unlike conventional trial-and-error approaches, AI-driven models can rapidly predict solvent properties, including CO_2_ absorption capacity, viscosity, regeneration energy, thermal stability, and cyclic performance using large experimental and computational datasets. Coupled with molecular simulations and high-throughput computational screening, these approaches substantially reduce experimental effort, development time, and research costs. Despite these advances, challenges remain regarding data quality, model interpretability, and the availability of standardized datasets. Future research should focus on integrating AI-driven solvent design with experimental validation and techno-economic optimization to accelerate the commercialization of next-generation CO_2_ capture technologies.

### 3.3 Direct air capture (DAC): emerging opportunities and challenges

Direct air capture (DAC) has emerged as an important negative-emission technology capable of removing CO_2_ directly from the atmosphere, where CO_2_ concentrations are approximately 420–430 ppm. Unlike conventional post-combustion capture systems, DAC employs highly selective liquid absorbents or solid sorbents to capture dilute atmospheric CO_2_ for permanent geological storage or utilization. Although DAC offers considerable potential for achieving net-zero emission targets and compensating for difficult-to-decarbonize sectors, its widespread deployment remains constrained by high energy consumption, significant capital investment, and elevated capture costs resulting from the low atmospheric concentration of CO_2_. Recent advances in low-energy absorbents, structured contactors, renewable energy integration, and process intensification have improved the technical feasibility of DAC systems. Nevertheless, further reductions in regeneration energy, process costs, and life-cycle environmental impacts are required before DAC can be deployed economically at large scale. Consequently, continued research integrating advanced absorbents, AI-assisted process optimization, and standardized techno-economic assessment will play a critical role in the future commercialization of DAC technologies.

## 4 Techno-economic evaluation of absorption-based CO_2_ capture systems

A coherent framework of assessing the practicality and general performance of the carbon capture technologies is provided by techno-economic analysis (TEA). It integrates engineering functionality and economic analysis, which allows a comprehensive insight into the functionality of a system in real-world situations. The main parameters such as the capital investment, operating and maintenance costs, energy requirement, and CO_2_ capture efficiency-are highly evaluated to determine the feasibility of the processes. Combining all these aspects, TEA enables researchers and industry experts to determine the best absorption methods, optimize the process, and find ways of reducing costs. Consequently, it has a critical part in the development of economically viable and environmentally friendly carbon capture solutions.^88^

As shown in Table 6, MEA-based capture generally lies at the higher end of reported cost ranges, whereas advanced amine blends, carbonate systems, ammonia-based processes, and hybrid membrane-absorption configurations can reduce capture cost under favourable assumptions but still exhibit significant variability due to differences in plant scale, integration, and economic context.

**Table 6:** Synthesised techno-economic comparison of absorption-based CO_2_ capture systems

S.No	Solvent/process configuration	Plant type & scale	Flue gas CO_2_ (vol%)	Integration/energy source	Cost metric reported	Capture cost range(USD per t CO_2_)
1	MEA (30 wt%)	Coal-fired power plant, ∼500 MW	12–15	Steam extraction from turbine; baseline design	Cost of CO_2_ captured/avoided	∼50–80
2	DEA/MDEA (conventional amines)	Natural gas processing/refinery units	5–25 (acid gas)	Process heat from gas plant	Cost of CO_2_ captured	∼35–65
3	Piperazine-promoted amine systems	Advanced post-combustion PCC, ∼400–500 MW	8–15	Enhanced heat integration; optimized stripper	Cost of CO_2_ captured	∼40–70
4	Hot potassium carbonate (HPC)	IGCC/syngas treatment	30–40	Syngas process heat	Cost of CO_2_ captured	∼30–60
5	Aqueous/chilled ammonia	Coal-fired plant flue gas, ∼500 MW	12–15	Steam plus refrigeration for chilled operation	Cost of CO_2_ captured	∼45–75
6	Amino acid salt/sterically hindered amines	Advanced absorption units, pilot scale	10–20	Partial heat integration	Cost of CO_2_ captured (estimate)	∼40–70
7	Ionic liquid/Novel solvent systems	Pilot-scale CCS/industrial flue gas	10–20	Various; often limited integration	Cost of CO_2_ captured (estimate)	∼60–90
8	Hybrid membrane + absorption	Post-combustion capture, ∼300–500 MW	12–15 (pre-concentrated to 20–25)	Steam plus electrical power for membranes	Cost of CO_2_ captured	∼40–70

Absorption-based CO_2_ capture, in turn, places economic assessment in a specific significance, as it will allow making a straightforward comparison with rival technologies. The cost analysis gives an understanding of the financial impact of implementing at large scale and assists in determining competitiveness of absorption processes. There are diverse economic evaluation tools used in TEA, which provide distinct views, benefits, and limitations as discussed in Table 7.^89,90^ Such methods can enable a more nuanced analysis of cost structures and performance trade-offs, to aid better and more strategic decision-making in designing and implementing carbon capture systems.

**Table 7:** Strengths and constraints of cost estimation methods in CO_2_ absorption technologies^89,90^

S.No	Economic estimator	Definition	Advantages	Limitations
1	Net present value (NPV)	Represents the sum of the present values of all cash flows, including the initial investment	• Provides a single value for economic analysis	• Sensitive to discount rate
			• Considers time value of money	• Requires accurate cash flow projections
			• Suitable for evaluating profitability over a relatively short lifespan	
2	Payback period	Defined as the duration required to recover the initial investment from revenues from the beginning of the project	• Simple to calculate and understand	• Ignores time value of money
			• Useful for quick assessment of risk	• Ignores cash flows after payback period
3	Internal rate of return (IRR)	Measures the difference between revenues and costs, discounting expenses and investment in relation to investment	• Considers time value of money	• Multiple IRRs may occur in some cases
			• Easy for non-financial stakeholders to understand	• Can be misleading for non-conventional cash flows
4	Levelized cost of CO_2_ capture	Indicates the average cost per unit of CO_2_ captured over the lifetime of the capture system	• Considers capital, operating, and maintenance costs	• Sensitive to assumptions about future costs and performance
			• Useful for comparing different technologies	• Oversimplifies complex costs into a single value
5	Combined TEA application	Use of NPV/IRR, payback, and levelized cost together for CO_2_ absorption projects	• Provides a more balanced view of profitability, risk, and cost competitiveness; supports fair comparison across solvents and configurations	• Requires transparent reporting of system boundaries, discount rates, energy prices, and uncertainty ranges

Table 7 highlights that NPV, IRR, payback period, and levelized cost of CO_2_ capture each emphasize different aspects of economic performance, and no single estimator is sufficient on its own to fully characterize absorption-based capture projects. Using these metrics in combination—while clearly stating key assumptions on discount rate, cash flows, and system lifetime—allows a more balanced evaluation of profitability, investment risk, and cost competitiveness for CO_2_ absorption technologies.

### 4.1 Methodological framework for techno-economic evaluation

The CO_2_ capture process can be divided into a set of interconnected steps, as shown in Fig. 8, which can be broadly grouped into technological analysis and economic evaluation. The technological stage entails the building of representative process model to establish mass and energy balances, especially with innovative or highly complex system. The modeling is normally performed with the help of process simulation software like DWSIM, Aspen Plus, or Aspen HYSYS that enable to analyze the behavior of the system in detail under specified operating conditions.^88^ Simulation provides an essential connection between the design and the use of concepts in the industry. It gives a guideline to make predictions on performance of the processes, assessment of various operating conditions and enables upscaling between laboratory or pilot systems to full deployment. Moreover, it assists in the calculation of the right equipment sizes and workload to achieve that the system could work effectively under the real production conditions and remain scalable. An inflexible techno-economic analysis, however, is highly dependent on the accuracy of equipment sizing and the accuracy of the cost estimate. Reliable techno-economic assessment requires accurate process modelling, equipment sizing, and transparent economic assumptions to ensure meaningful evaluation of carbon capture technologies.

**Fig. 8:**
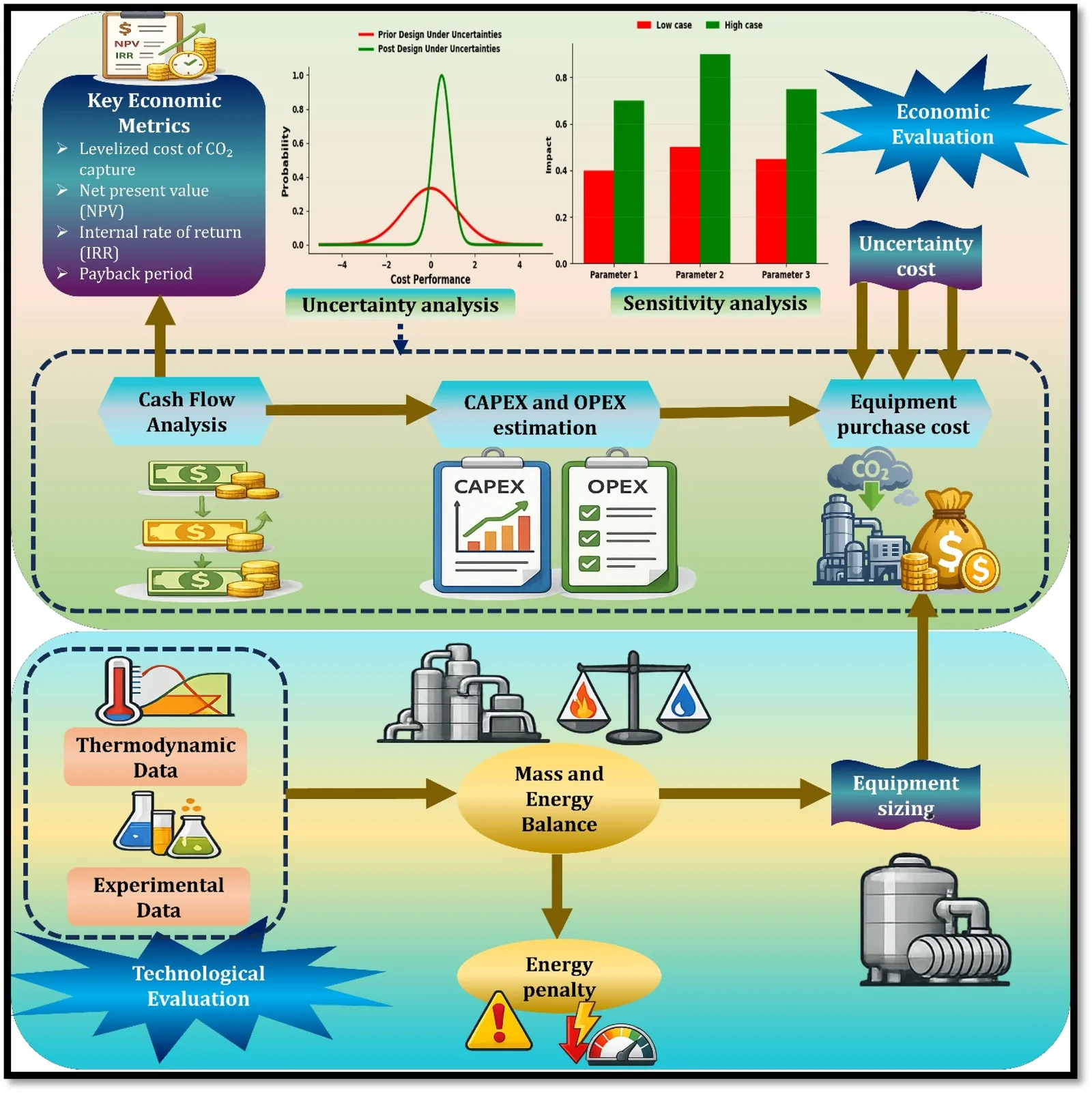
Systematic approach to techno-economic assessment of absorption-based carbon capture processes.

The process design in terms of techno-economic analysis requires the strict analysis of thermodynamic properties, behavior of transport, and reaction kinetics.^89^ Thermodynamics determines the theoretical limits of the system such as equilibrium states, maximum conversion and energy needs all of which have a direct bearing on viability and efficiency of the process. On the other hand, the rate at which processes occur and the size and arrangement of equipment required, such as reactors, heat exchangers and separation equipment are dependent on the kinetic parameters and transport processes such as heat and mass transfer. So, it is important to be able to depict these factors properly and in a unified way so that process simulations and the consequent analyses would be reliable.^90^

After proper sizing of the equipment, the second step is to estimate capital costs. It may be done with the help of specific software, such as CAPCOST, proposed cost correlations based on technical literature, or commonly known design references such as Peters and Timmerhaus. Moreover, Aspen Capital Cost Estimator, which is a commercial platform, offers systematic procedures of cost analysis in a detailed way.^91,92^

In cases where equipment capacity falls outside the range of data or tools applicable, the cost estimation may be extrapolated by use of information available in published literature or vendor lists, frequently with scaling techniques like the six-tenths-factor rule. Since data on the cost of equipment is often based on previous time periods, there is need to update such values to reflect the prevailing economic conditions. This is usually done by the use of such cost indices as the Chemical Engineering Plant cost index (CEPCI), which can be used to estimate costs consistently and time adjusted.^93^

To improve consistency among published studies, techno-economic assessments of carbon capture systems should follow standardized evaluation methodologies that clearly define system boundaries, functional units, economic assumptions, plant lifetime, discount rate, capacity factor, and energy pricing. Guidelines proposed by organizations such as the IEAGHG recommend transparent reporting of capital expenditure, operating expenditure, CO_2_ capture efficiency, energy penalty, and cost of CO_2_ avoided to facilitate meaningful comparison among competing technologies. Furthermore, uncertainty and sensitivity analyses should accompany all techno-economic studies to quantify the influence of key parameters, including fuel price, solvent degradation, electricity cost, and carbon pricing, on the overall economic performance of CCS systems. Adoption of standardized assessment methodologies would improve the reliability, reproducibility, and comparability of future techno-economic evaluations.

Once the technological evaluation is done, the next step is the economic evaluation whereby the capital expenditure (CAPEX) and operating expenditure (OPEX) is systematically estimated. These cost elements are then applied to carry out financial analysis relying on discounted and non-discounted cash flow methods, which allow calculating the key economic performance indicators, as in Table 5. In order to establish a complete picture of the effect that input assumptions have on the overall viability of the process, sensitivity and uncertainty analysis is usually performed.^94^ These techniques can be used to analyze the impacts of changes in key parameters on economic outcomes and, therefore, to determine the most significant variables and reveal the possible risks of uncertain data. These insights are critical towards enhancing the credibility of the assessment, risk management and informed investment decision making. Sensitivity analysis uses one input parameter at the time, holding all other parameters constant, and thus it is feasible to isolate and estimate the effect of a single variable on model outputs.^95^ This is especially effective in identifying which variables have the most significant impact on the performance of the economy. Uncertainty analysis, in contrast, takes into account the concurrent variability of several inputs and assesses the resultant distribution of potential outcomes, often probabilistically. This gives a wider view of the scope of possible circumstances and probability of various outcomes. Both analyses have more detailed methods presented in the corresponding literature.^96,97^

### 4.2 Recent advances in techno-economic evaluation of absorption-based CO_2_ capture

Despite the evident popularity of techno-economic analysis of CO_2_ capture technologies, relatively few studies have performed cost-analysis of specific technologies (absorption-based) in detail. When used at a large scale, the necessity to handle large amounts of gas requires much bigger equipment to be deployed. This, on its part, increases capital investment, especially of heavy-duty infrastructure and high-capacity solvent systems. Instead of enjoying the traditional economies of scale, these systems can experience diseconomies of scale, in which the costs grow as they get larger.^98^ Additionally, operating and maintenance costs are exacerbated by the increased thermal energy needed to regenerate larger inventories of solvents, increasing the total economic impact.

Reported CO_2_ capture costs vary considerably across published studies, ranging from approximately 25.7 to 74 USD per t CO_2_, primarily because of differences in system boundaries, plant capacity, solvent type, energy integration strategy, capture efficiency, economic assumptions, and regional energy prices. Consequently, direct comparison of reported costs without considering these factors may lead to misleading conclusions regarding technology performance. Large-scale commercial facilities generally benefit from economies of scale, whereas pilot-scale studies often report higher unit capture costs because of limited process integration and higher specific capital investment. Similarly, advanced solvent systems that exhibit lower regeneration energy do not necessarily result in lower capture costs if solvent replacement, degradation, or equipment costs increase.

The analysis of mini-channel absorbers as an alternative perspective takes into consideration both the capital and operating costs by taking the net present value. According to these studies, mini-channel configurations may be cost-effective in some circumstances, especially when the scale of a plant is small.^99^ Reported cost savings in facilities with capacities between 5 and 50 MMSCFD range between about 50% at lower capacities to about 3% at higher capacities, mostly attributed to a low initial capital investment. But with the system's capacity, the comparatively high operating costs of mini-channel designs are likely to override their initial economic advantages, thus affecting their competitiveness in comparison to traditional absorption systems. A case study of the implementation of carbon capture and sequestration (CCS) at the Hunter Power Plant of PacifiCorp further adds to the discussion of how scale affects the performance of economic activity. The paper has discussed Unit 3 which burns low-sulfur sub-bituminous coal and evaluated performance and cost-effectiveness of incorporating the MHI KM-CR Processed KS-1-solvent. The findings suggest that capture costs decrease with the increase in the capture rate. Approximately 50, 61 and 74 USD per ton of CO_2_ were estimated to capture 90, 65, and 1000 lbs/MWh-g, respectively, and achieve CO_2_ purity greater than 95. These results emphasize the importance of the scale of the system and optimization of its operation to enhance the cost-effectiveness of CCS implementation.^100^

Another techno-economic analysis compared post-combustion capture of CO_2_ by co-purifying a standard monoethanolamine (MEA) system and a newly developed absorbent to a power plant at dynamically varying conditions.^101^ These findings revealed that the MEA-based process has a capture cost of approximately, 35.5 dollars per ton of CO_2_ in the Republic of Korea mainly because of its rather high solvent regeneration energy demand which is approximately 3.5 GJ per ton of CO_2_. Comparatively, the new absorbent had a significantly lower regeneration energy requirement of 2.17 GJ per ton of CO_2_ which reduced the capture cost by about 25.7 per ton, thus showing a superior economic performance. The same was also done with post-combustion CO_2_ capture using aqueous piperazine as the process efficiency was determined as a function of flue gas CO_2_ concentration, gas flow rate and total capture efficiency. The research indicated that the higher the inlet CO_2_ concentration, the better the performance of the system. It is worth noting that the optimum capture efficiency was 95% at an inlet concentration level of 33 vol% CO_2_, which was the lowest capture cost. The results highlight the importance of flue gas composition in the optimization of technical and economic performance of CO_2_ capture technologies.

Different absorption-based CO_2_ capture ideas have proven to have a considerable cost advantage over traditional techniques, and thus are appealing technologies which can be implemented on a large scale in the future. Nevertheless, most of the current studies are small scale, either in the laboratory or pilots, which validates the need to verify the economic performance in a more stringent environment in the conditions of industrial functioning. A new pre-combustion capture system suggested combines a new solvent looping idea into steam methane reforming (SMR) systems. Such a design greatly decreases the thermal power needed to regenerate solvents especially by lowering reboiler duty in comparison with typical post-combustion processes. Blended methyldiethanolamine (MDEA) and piperazine (PZ) solvents techno-economic analysis shows that the unit price of the CO_2_ captured decreases with the capture efficiency.^102^ The energy consumption and overall costs of operation in the system are also reduced.

Simultaneously, Siefart *et al.* proposed two hydrophobic physical solvents PEG-Siloxane-1 and [aPy][Tf2N] that were aimed at removing CO_2_ in pre-combustion syngas streams in integrated gasification combined cycle (IGCC) plants. Using Aspen Plus simulations combined with detailed economic modeling, the levelized cost of CO_2_ capture (LCOC) was assessed.^103^ These results indicate that PEG-Siloxane-1 is as effective as Selexol, a standard commercial solvent, and the financial viability of [aPy][Tf2N] will depend on its scalability, and its cost of production at industrial scales.

Another study by Kotamreddy *et al.* was on the microencapsulated carbon capture solvents (MECS) that were prepared with sodium carbonate solutions. Their process model and economic analysis indicated that optimization of residence time could result in smaller reactor size and that heat integration can be highly efficient in reducing the energy demands of regeneration is very necessary.^104^ Regardless of these benefits, MECS-based fixed-bed systems were observed to have an equivalent annual operating cost (EAOC) that was 1.8 to 2.7 times greater than that of more traditional Monoethanolamine (MEA) systems with high heat recovery. Continued advances in capsule technology and contactor design should increase the competitiveness of this technology.

Direct air capture (DAC) is becoming more popular as a potential solution to dispersed CO_2_ emissions, which are difficult to capture at point sources. Barzagli *et al.* carried out a study to examine the performance of different alkanolamines that are usually utilized in carbon capture. It was found that the capture efficiencies of aqueous primary unhindered amines can be as high as those of alkali hydroxides, and the regeneration energy requirements may be lower. It was found that the amine carbamates formation and stability are the determinants of the capture effectiveness.^105^

More so, hybrid designs comprising of membrane separation and enzymatic absorption have also been suggested to improve overall system performance. In a 600 MWe power plant application, an amine-based absorption process was combined with membrane technology, to eliminate the high energy and capital requirements of independent systems.^106^ The results of the optimization showed that 124 MW of electricity and 124 MW of annual operating cost were reduced relative to a membrane-only installation (by 10 and 37%, respectively). Such a combined solution is more energy efficient and cost-benefitable compared to the traditional CO_2_ capture systems.

## 5 Challenges and roadmap for future development

Absorption-based carbon capture technology has been identified as a promising approach for reducing greenhouse gas emissions due to continuous advancements in CO_2_ separation techniques. However, its perceived maturity often obscures persistent systemic limitations that hinder large-scale industrial deployment. Several critical challenges must still be addressed before widespread commercialization can be realized. These limitations require more systematic and critical evaluation to guide future development toward practical implementation.

### 5.1 CO_2_ capture *vs.* net CO_2_ reduction

Absorption processes can remove a large fraction of CO_2_ from flue gas streams, but the net reduction in emissions depends on the additional energy required for solvent regeneration and on upstream emissions associated with solvent production and plant integration. The energy penalty reduces overall plant efficiency and can offset part of the captured CO_2_, particularly when regeneration heat is supplied by fossil-based energy. This makes it essential to couple absorption-based capture with low-carbon energy sources, effective heat integration, and process intensification strategies when assessing the real climate benefit of CO_2_ capture schemes rather than relying solely on capture efficiency.

### 5.2 Solvent design, environmental footprint, and waste handling

One of the key factors influencing process performance is the selection and design of appropriate solvents. Absorption systems depend on solvents that exhibit high CO_2_ selectivity and affinity, low toxicity, minimal environmental impact, and high operational stability. Achieving an optimal balance among these competing properties remains a fundamental challenge, often involving trade-offs between reactivity, energy demand, and long-term stability. Continuous innovation in solvent design must therefore be accompanied by careful assessment of solvent degradation products, emissions, and toxicity, as well as the environmental footprint of solvent manufacture and disposal. Solvent degradation and loss are particularly important because they affect both performance and environmental outcomes. During continuous operation, solvents are exposed to CO_2_ and impurities, leading to chemical degradation that alters physicochemical properties, reduces CO_2_ absorption capacity, and decreases overall process efficiency. This necessitates increased solvent replacement and more intensive regeneration, raising operating costs through chemical consumption, waste management, and energy use. In addition, evaporative losses, especially of volatile amines and ammonia, can generate solvent emissions that pose environmental and health concerns and require appropriate capture, treatment, or regulatory controls. Effective solvent management-including monitoring, recovery, recycling, and proper waste handling is therefore crucial not only for economic viability but also for minimizing environmental impacts. Because during continuous operation, solvents are exposed to CO_2_ and impurities, leading to chemical degradation that alters physicochemical properties, reduces CO_2_ absorption capacity, and decreases overall process efficiency.^107^

### 5.3 Materials gaps and process gaps

From a materials perspective, a key gap is the absence of solvents that simultaneously offer low regeneration energy, high cyclic stability, low volatility, and minimal toxicity under realistic flue gas conditions. Many emerging systems improve one or two attributes—such as lowering energy demand or enhancing stability—while introducing new issues related to viscosity, precipitation, corrosiveness, or environmental risk. From a process perspective, major gaps include limited deployment of advanced heat integration and compact contactor designs, incomplete treatment of solvent degradation and management in techno-economic assessments, and a lack of standardized methodologies for comparing capture configurations across different plants and solvent systems. Addressing both materials and process gaps in a coordinated way is essential to move absorption-based CO_2_ capture beyond incremental improvements toward genuinely optimized and deployable technologies.

### 5.4 Energy consumption, process intensification, and hybrid systems

Energy consumption remains one of the most critical constraints, especially in chemical absorption systems where solvent regeneration is the most energy-intensive step. In conventional stripper units, solvent regeneration can account for a substantial portion of the total capture energy input, significantly reducing net plant efficiency and, in some cases, undermining the environmental benefits of CO_2_ capture. This underscores the need for improved absorbents, enhanced heat integration strategies, and alternative low-energy regeneration routes. Process intensification approaches—such as absorber intercooling, advanced stripper configurations, structured packings, and compact or modular contactors—can reduce equipment size and regeneration duty, but they often introduce additional control and design complexity that must be carefully managed. Hybrid systems, particularly those combining membranes with absorption (for example, using a pre-concentrating membrane upstream of a solvent unit), can lower solvent circulation rates and reboiler duty by increasing flue gas CO_2_ concentration before absorption. However, such systems also incur additional compression work, power consumption, and capital costs for membrane modules, making it essential to evaluate their net benefit through integrated techno-economic and environmental assessments rather than unit-level performance alone.

### 5.5 Quantitative energy and cost perspective

Given the central role of energy in determining feasibility, future work should present more quantitative breakdowns of energy and cost. Typical absorption-based post-combustion capture systems rely on regeneration duties that can represent a major fraction of the total capture energy requirement and cause noticeable efficiency penalties at the plant level. Capture costs are strongly influenced by this energy demand, solvent make-up, degradation management, and integration strategies. It is therefore important that techno-economic evaluations report key indicators such as regeneration energy, net efficiency loss, and levelized capture cost using consistent system boundaries, as well as sensitivity analyses for major economic assumptions. Such quantitative reporting will enable meaningful comparison among baseline MEA systems, advanced solvent formulations, intensified absorption processes, and hybrid membrane-absorption configurations, and help identify which combinations of materials and process designs are most promising for achieving cost and energy targets required for large-scale deployment.

Ultimately, future progress will depend on integrating material innovation with process intensification, solvent management, and realistic techno-economic and environmental assessment. By jointly addressing solvent properties, energy use, environmental footprint, and process configuration, absorption-based CO_2_ capture can be advanced toward more efficient, cost-effective, and sustainable carbon capture systems capable of supporting long-term decarbonization goals.

## 6 Conclusions

This review critically evaluated the current status of absorption-based CO_2_ capture technologies by examining solvent development, process intensification strategies, desorption enhancement techniques, and techno-economic assessment approaches. In particular, it integrated these aspects into a unified perspective that links solvent behaviour directly to process energy penalties and cost metrics, moving beyond purely material-centric discussions. Among the various absorbents, conventional amine-based solvents, particularly monoethanolamine (MEA), remain the industrial benchmark because of their high CO_2_ absorption efficiency, well-established process design, and commercial maturity. However, their widespread deployment continues to be constrained by high regeneration energy requirements, solvent degradation, equipment corrosion, and significant operating costs. Emerging solvent systems, including blended amines, amino acid salts, phase-change solvents, ionic liquids, and deep eutectic solvents, demonstrate considerable potential to reduce energy consumption and improve cyclic stability, although most have only been validated under laboratory or pilot-scale conditions. The analysis in this review underscores that, despite these advances, no single solvent system yet satisfies the combined requirements of low energy demand, high stability, low environmental impact, and favourable economics under realistic industrial operating conditions.

From a techno-economic perspective, the largest barrier to large-scale implementation remains the high energy demand associated with solvent regeneration, which substantially increases operating expenditure and overall CO_2_ capture costs. In addition, solvent replacement, process integration, capital investment, and uncertainty in economic assumptions contribute significantly to variations in reported capture costs across different studies. Therefore, future techno-economic evaluations should adopt standardized methodologies with clearly defined system boundaries, sensitivity analyses, and transparent reporting of economic assumptions to enable meaningful comparison among competing technologies. A key contribution of this review is the proposal of a structured solvent comparison and TEA framework that explicitly incorporates degradation, solvent management, and desorption efficiency into cost and energy assessments, providing a more process-relevant basis for comparing absorption-based capture systems.

Future research should focus on developing low-energy, chemically stable, and environmentally sustainable absorbents while integrating advanced process intensification strategies and renewable energy sources to reduce overall capture costs. Greater emphasis should also be placed on long-term pilot-scale demonstrations, solvent stability under realistic flue gas conditions, life-cycle assessment, and standardized techno-economic evaluation frameworks to accelerate the commercial deployment of next-generation absorption-based CO_2_ capture technologies. Informed by the gaps identified in this review, priority should be given to integrated studies that co-optimise solvent formulation, regeneration strategy, and economic performance rather than treating these elements in isolation. Such integrated efforts will play a crucial role in enabling economically viable and sustainable carbon capture systems that support global decarbonization goals.

## Conflicts of interest

There is no conflicts to declare.

## Data Availability

No primary research data were generated or analysed in this review. All information and data discussed in this article are derived from the cited literature.
